# Mindfulness-Based Restoration Skills Training (ReST) in a Natural Setting Compared to Conventional Mindfulness Training: Psychological Functioning After a Five-Week Course

**DOI:** 10.3389/fpsyg.2020.01560

**Published:** 2020-08-12

**Authors:** Freddie Lymeus, Marie Ahrling, Josef Apelman, Cecilia de Mander Florin, Cecilia Nilsson, Janina Vincenti, Agnes Zetterberg, Per Lindberg, Terry Hartig

**Affiliations:** ^1^Department of Psychology, Uppsala University, Uppsala, Sweden; ^2^Institute for Housing and Urban Research, Uppsala University, Uppsala, Sweden

**Keywords:** mindfulness, restoration, meditation, nature, environment, training, psychological functioning

## Abstract

Restoration skills training (ReST) is a mindfulness-based course that draws on restorative nature experience to facilitate the meditation practice and teach widely applicable adaptation skills. Previous studies comparing ReST to conventional mindfulness training (CMT) showed that ReST has important advantages: it supports beginning meditators in connecting with restorative environmental qualities and in meditating with less effort; it restores their attention regulation capabilities; and it helps them complete the course and establish a regular meditation habit. However, mindfulness theory indicates that effortful training may be necessary to achieve generalized improvements in psychological functioning. Therefore, this study tests whether the less effortful and more acceptable ReST approach is attended by any meaningful disadvantage compared to CMT in terms of its effects on central aspects psychological functioning. We analyze data from four rounds of development of the ReST course, in each of which we compared it to a parallel and formally matched CMT course. Randomly assigned participants (total course starters = 152) provided ratings of dispositional mindfulness, cognitive functioning, and chronic stress before and after the 5-week ReST and CMT courses. Round 4 also included a separately recruited passive control condition. ReST and CMT were attended by similar average improvements in the three outcomes, although the effects on chronic stress were inconsistent. Moderate to large improvements in the three outcomes could also be affirmed in contrasts with the passive controls. Using a reliable change index, we saw that over one third of the ReST and CMT participants enjoyed reliably improved psychological functioning. The risk of experiencing deteriorated functioning was no greater with either ReST or CMT than for passive control group participants. None of the contrasts exceeded our stringent criterion for inferiority of ReST compared with CMT. We conclude that ReST is a promising alternative for otherwise healthy people with stress or concentration problems who would be less likely to complete more effortful CMT. By adapting the meditation practices to draw on restorative setting characteristics, ReST can mitigate the demands otherwise incurred in early stages of mindfulness training without compromising the acquisition of widely applicable mindfulness skills.

## Introduction

Restoration skills training (ReST) is a mindfulness-based meditation training course given in a setting rich in natural features and processes (Lymeus et al., 2018, 2019). ReST thus integrates two complementary approaches to helping people who struggle to manage the demands in their lives: one based in clinical and health psychology that employs a method for individual training to strengthen adaptive capabilities (see e.g., Bishop et al., 2004), and one based in restorative environments research that builds on the provision of contextual support for periodic replenishment of adaptive capabilities (see e.g., Hartig et al., 2011). The development of ReST has had two overarching aims: (1) it should help beginning meditators build a meditative state on restorative experiences in a natural setting, and (2) it should confer generalized benefits for psychological functioning at least similar to those of conventional mindfulness training (CMT). Previous studies (Lymeus et al., 2018, 2019) have yielded findings indicating that ReST satisfies the first aim. This article addresses the second aim, with a view to benefits in terms of improved dispositional mindfulness, improved cognitive functioning, and reduced chronic stress.

In the following, we first outline relevant theory and issues in mindfulness research. Second, we outline how we connect mindfulness with restorative environments theory. Third, we describe the development of the practice approach of ReST. Fourth, we summarize the previous findings on the efficacy of ReST. Fifth, we specify our aims and hypotheses regarding the benefits achieved with the two approaches, tested in the empirical work reported in the subsequent sections.

### Mindfulness Training

Mindfulness is a concept derived from Buddhist philosophy that refers to a particular quality of awareness that arises when someone intentionally attends to present experience and withholds judgments and reactions to it (Kabat-Zinn, 1994; cf. Lutz et al., 2007; Grabovac et al., 2011; Eberth and Sedlmeier, 2012). For an operational conception of mindfulness, we have built mainly on the definition proposed by Bishop et al. (2004). They describe mindfulness as entailing attentiveness to present experience with qualities of curiosity (i.e., experiential openness and acceptance) and decentering (i.e., viewing one’s thoughts and feelings about experience as subjective and transient).

Mindfulness can be construed and observed in several ways (see e.g., Berkovich-Ohana and Glicksohn, 2014; Davidson and Kaszniak, 2015; Lutz et al., 2015). Phenomenologically, mindfulness can be seen as an experiential state that varies in prominence as people go through different environments and activities (including but not limited to meditation). Mindfulness can also be seen as a cognitive-behavioral practice that people can choose to engage in to alter their relationship with experiences, as in meditation or in the application of mindfulness techniques in daily life situations. Mindfulness can additionally be seen as a personal disposition, or a general tendency to relate mindfully to experiences in many situations. Finally, mindfulness can be seen as a form of training in which people can invest through regular practice that over time will enhance their dispositional mindfulness and other aspects of psychological functioning. Approached in each of these ways, mindfulness can be related to adaptive capability and, ultimately, health outcomes. Our main focus here, however, is on mindfulness training.

#### Practices and Processes

In pursuing mindfulness training over time, regular meditators presumably progress through different stages in which different types of practices align with different skill-levels and needs (Lutz et al., 2008, 2015). In the early stages of mindfulness training, the emphasis is typically on practices aimed to improve attention regulation capabilities (Lutz et al., 2008; Tang et al., 2015). This aim is rooted in tradition and has been absorbed into contemporary theory (Lutz et al., 2007; Grabovac et al., 2011; Sedlmeier et al., 2012). In considering how attentional improvements might come about, Tang, Posner, and colleagues (Tang and Posner, 2009; Posner et al., 2010) distinguish between attention network training and attention state training. In attention network training, a person engages in repetitive practice with the aim to stimulate enhancements of the involved neural networks. This is done in so-called focused-attention exercises, in which participants try to sustain attention to a given target stimulus, such as sensations arising with the breath, and repeatedly redirect attention when they inevitably become distracted (Hasenkamp et al., 2012; Lutz et al., 2015). The practice is thought to require considerable effort from beginning practitioners (Lutz et al., 2008; Tang et al., 2015).

In contrast to attention network training, attention state training occurs when a person learns to draw on ongoing experience to balance their bodily and mental state to gain better access to existing attentional functions (Tang and Posner, 2009; Posner et al., 2010). This is mainly done in open-monitoring exercises, in which participants allow attention to rest on any aspect of experience that becomes salient while they try to remain mindful of their attentional shifts and the changing contents of consciousness (Bishop et al., 2004; Lutz et al., 2008, 2015). Such practice is thought to promote reflexive awareness of the subject-object continuum, which in turn can serve a sense of oneness with the surrounding world and, ultimately, insight. Open-monitoring is less effortful than focused-attention practice (Lutz et al., 2015; Fox et al., 2016). However, open-monitoring is commonly thought to be difficult for beginners who lack in ability to balance effortlessness with attentional stability. The established view is that beginners should first train their attention regulation capabilities through focused-attention exercises.

Congruent with an attention network training rationale, most mindfulness courses for beginners emphasize focused-attention practices. These courses typically span several weeks up to a few months during which participants attend classes that entail guided meditation as well as theoretical discussions (Carmody and Baer, 2009; Eberth and Sedlmeier, 2012; Davidson and Kaszniak, 2015; cf. e.g., Kabat-Zinn, 1990; Segal et al., 2002). Most courses also involve instructions to practice regularly with the given meditation exercises between the classes and consultation on how to establish a regular meditation habit.

#### Issues in Research and Implementation

Evidence reviews show that beginning mindfulness training improves several aspects of attention regulation and related adaptive capabilities (Brown et al., 2007; Chiesa et al., 2011; Gallant, 2016) and alters some relevant structures and functional patterns in the brain (Fox et al., 2014, 2016; Tang et al., 2015). However, the strength of the evidence for mindfulness training suffers from heterogeneity and vague descriptions of the training methods (Davidson and Kaszniak, 2015; Van Dam et al., 2018). Many studies refer to mindfulness training generically without further description of the course contents or specific practices used. One exception is the well-described and extensively studied Mindfulness-Based Stress Reduction (MBSR) course (Kabat-Zinn, 1990, 2011). Two meta-analytic reviews by Sedlmeier and colleagues (Eberth and Sedlmeier, 2012; Sedlmeier et al., 2018) show that in studies comparing MBSR for non-patient populations to passive control conditions, the average benefit across a range of psychological health outcomes is medium sized (in studies published before 2011, *r* = 0.31, and in studies published 2011–2015, *r* = 0.22). However, MBSR involves a mix of focused-attention, open-monitoring, yoga, and compassion practices which may work through different mechanisms.

The evidence also suffers from difficulties in finding and implementing appropriate comparison conditions for evaluating the effects of mindfulness-based interventions (Eberth and Sedlmeier, 2012; Davidson and Kaszniak, 2015). Few studies have used feasible active control conditions and experimental contrasts between different approaches to mindfulness training are rare. With inadequate description and poor experimental control, it is difficult to determine the merits of different practice approaches and it remains uncertain whether attention network training through focused-attention practice is necessary to achieve the desired benefits.

Most research on mindfulness (as well as other heath interventions) rely on group-based statistical approaches to evaluation. To evaluate the practical utility of a health intervention, however, several authors have argued for evaluating outcomes on the individual level, in terms of how many of the participants enjoy improvement (Howard et al., 1996; Chambless and Hollon, 1998). In principle, a group’s average change can be reliably positive with a non-trivial effect size even if few of the participants experienced any substantive improvement (Jacobson and Truax, 1991). Indeed, a group’s average change can also be reliably positive even if many of the participants actually experienced deterioration. However, few mindfulness studies have reported negative effects (Van Dam et al., 2018). Baer et al. (2019) recently reviewed the literature for reports of harm in mindfulness studies and concluded that transient discomfort, including that associated with effort, may be part of the process in some instances. Undesired change can also occur for external or endogenous reasons that coincide with the training; however, they may still be important to evaluate. Serious adverse events (i.e., suicidal behavior, psychiatric hospitalization) have been reported on rare occasions while symptom deterioration tends to occur for up to 10% of participants in those studies that have reported negative outcomes. However, Baer et al. (2019) note that the risk of deterioration with mindfulness training generally equals that seen in passive control conditions. To determine individual-level change, different approaches are available. One approach is to evaluate individual change against a criterion for a minimal important difference: a construct used in clinical research to represent the smallest difference in a health-related outcome that would be perceived as meaningful by patients and clinicians (Revicki et al., 2008). Another is the statistical approach of the reliable change index (RCI) proposed by Jacobson and Truax (1991).

A problem that may be related to transient discomfort or longer-lasting deterioration is that a substantial proportion of participants drop out or comply poorly with the intended training program in conventional mindfulness courses. In their meta-analytic review of attrition in randomized controlled mindfulness-based treatment studies, Nam and Toneatto (2016) found an average drop-out rate of 29% (range 5–63%). Sekhon et al. (2017) describe compliance as a reflection of the acceptability of a psychological intervention. Poor acceptability in some subgroups of the target population can be a major problem in evaluations and in practice (Hollon et al., 2002; Nam and Toneatto, 2016). Given that attentional enhancements have been considered as central to achieving generalized functional improvements through mindfulness training, the issue of acceptability is a major concern with people who enter into training with attention regulation difficulties; they seem to be particularly unlikely to complete the courses and the recommended amounts of homework exercise (Crane and Williams, 2010; Lymeus et al., 2017; Banerjee et al., 2018). Those participants who have the most to gain from a mindfulness training course might therefore be least likely to complete the training. A means to achieve comparable benefits for these people without imposing further strain on their weak attention regulation capabilities could meaningfully improve the practical effectiveness of mindfulness training.

### Connecting Mindfulness and Restorative Environments Theory

#### Complementarity in Processes

In different Buddhist traditions, meditation in nature has deep historical and spiritual roots (Fisher, 2007, 2014; Coleman, 2010; Mahanta and Campus, 2010; Fabjański and Brymer, 2017; Van Gordon et al., 2018). However, contemporary research has mainly studied meditation as an intra-individual practice, neglecting contextual factors. In the following, we outline how restorative environments research, and most notably the attention restoration theory (ART) introduced by Kaplan and Kaplan (1989), Kaplan (1995), can serve understanding of the role of the setting in mindfulness. We also indicate how mindfulness theory can inform restorative environments research.

Attention restoration theory is commonly used to explain how access to nature can help people who struggle to focus and manage demands in their usual living and working conditions within modern urbanized societies (Kaplan and Kaplan, 1989; Kaplan, 1995; Hartig et al., 2011; Hartig and Kahn, 2016). With undemanding activities in natural versus indoor or built outdoor settings, overworked attention regulation capabilities can be regained and stress can be reduced more readily (for reviews of the evidence, see Hartig et al., 2014; Ohly et al., 2016; Stevenson et al., 2018). ART holds that when people leave their normal activities and enter a setting that is rich in natural features and processes, they can gain a sense of psychological detachment from demands and routine mental contents, an experiential quality called being away (Kaplan and Kaplan, 1989; Kaplan, 1995). They can also engage with pleasantly interesting aspects of the environment that draw attention effortlessly but not forcibly, an experiential quality called soft fascination. Although little research has studied the progression over time in extended visits, Kaplan and Kaplan (1989) theorize that a person will first tend to experience a clearing of the head from residual fragments of what they just left behind. Then the person will start to regain the capacity to regulate attention and act more deliberately. After that, the person will gain cognitive quiet as their normal concerns settle. Finally, the person may find room for deep reflection that draws towards a sense of oneness with the natural world. The later stages identified by Kaplan and Kaplan are akin to the processes thought to be involved in open-monitoring practice, including reflexive awareness and insight (cf. Lutz et al., 2008, 2015).

With its focus on processes and outcomes in individual instances of nature contact, research founded in ART is most clearly connected to the branches of mindfulness research that are based in the state and practice views of the phenomenon. In the development of ReST, we have drawn conceptual links between mindful curiosity and soft fascination, and between mindful decentering and the sense of being away (Lymeus et al., 2018, 2019; Lymeus, 2019). Of more concern here, however, we have also recognized the potential that lies in their differences. The mindfulness literature typically sees attentiveness, curiosity and decentering as personal capabilities that are exerted by will. In contrast, restorative environments theory sees soft fascination and being away as transactional phenomena that can arise in the spontaneous exchange between a person and the environment, and the enhanced capacity for willful regulation of attention as an outcome of such transactions. Meditation in nature could therefore momentarily support state mindfulness even when a person lacks in capability to self-regulate the involved processes. With regular meditation in a course of training over time, however, the attention network training rationale would predict that enhanced capability for mindfulness across many situations would come with the regular expenditure of effort, so a greater ease of practice should blunt the benefits. Effortless training would therefore require another mechanism for accumulation of benefits.

#### Accumulation of Benefits

Much as with mindfulness training, a long-standing assumption in restorative environments research is that the benefits of nature contacts can accumulate in individuals over time when they access nature repeatedly (see Hartig et al., 2014). Such cumulative effects have been surmised for example from findings of better psychological functioning and health among people who have relatively much green space near their home (e.g., Kuo and Sullivan, 2001; Maas et al., 2006; Mitchell and Popham, 2008; Sugiyama et al., 2008; Ward Thompson et al., 2012), or who regularly engage in extended nature visits (White et al., 2019). However, restorative environments research has predominantly assumed that mere repetition of restoration patterns over time sustains psychological functioning and, ultimately, health (e.g., Hunter et al., 2019; White et al., 2019). With that assumption, the progress has been limited in understanding the learning processes that may be involved and how restorative experiences might shift in quality and meaning with repeated exposures. One notable exception is Kaplan (2001), who draws in part on meditation literature to indicate how people might learn from experience to notice needs for restoration as well as where and how they can draw restorative benefits efficiently in order to enhance adaptation over time. These notions are akin to what Tang and Posner (2009) describe as attention state training, and are at the core of the ReST approach to mindfulness training.

### The ReST Approach to Mindfulness Training

As a starting point for the development of ReST, we took the well-established MBSR course (Kabat-Zinn, 1990). In an initial study, Lymeus (2008; later published as Lymeus et al., 2017) administered attention tests before and after meditation exercises, with repeated assessments over 8 weeks of training in MBSR. Between the completion of the study in 2008 and its publication in 2017, considerable development took place in the theoretical and neurophysiological understanding of how effortful processes become engaged in early stages of CMT (see Lutz et al., 2008, 2015; Tang and Posner, 2009; Hasenkamp et al., 2012; Malinowski, 2013; Tang et al., 2015; Fox et al., 2016). However, the Lymeus et al. (2017) publication was, to our knowledge, the first to report performance test data that specifically targets the short-term effects of effort and restoration involved in mindfulness exercise. It showed that, over the eight course weeks, conventional mindfulness exercises incurred increasing effort, as reflected in deteriorated attentional test performance. Presumably, the participants gradually learned the skills needed to regulate the mindful state through attentional effort. In contrast, participants who meditated with nature images had increasingly positive change in test performance over the weeks and improved towards the end of the course. Apparently, as their mindfulness skills improved, they learned to draw support for their meditation from the natural stimuli.

Following those findings, we set out to adapt the training approach in MBSR to draw deliberately on restorative qualities in a natural meditation setting. To accomplish this, the practice approach built on open-monitoring and deemphasized willful attention regulation. Instead, we assumed that restorative environmental qualities would help the beginning meditators by drawing attention to present experiences in the environment, stimulating curiosity, facilitating decentering, and restoring attention regulation capabilities. Compared to the most common MBSR format, ReST uses a briefer, 5-week format and shorter class (90 min) and exercise (ca. 20 min) durations (which is in line with multiple other adapted mindfulness training approaches; e.g., Linehan, 1993; Tang et al., 2007; Zylowska et al., 2008; also see Carmody and Baer, 2009). Like MBSR, ReST retains a progressive structure where each subsequent course week introduces new concepts and practices that build on the preceding weeks’ content. We developed the weekly themes and the specific contents over four data collection rounds (see Lymeus, 2019 for additional detail on the development process). In each round, we also updated CMT to formally match ReST.

In the first data collection round, ReST course contents were quite similar to CMT but placed more emphasis on experiences emanating from contacts with the environment. Building on preliminary evaluations of instructor and participant experiences and data obtained in that round (see Florin and Lundström, 2013 [Lundström n.k.a. author MA]), we considered that some further adaptations of the practice approach could likely support participants in engaging with the environment in a more beneficial manner. In the second data collection round, we therefore dedicated each course week to exploration of experience through a specific sensory modality. We saw room for further improvement (see Apelman, 2013) which, in the third round, led us to develop the instructional language and the connections between the exercises and the theoretical themes. Our preliminary evaluations indicated that we had handled most of the practical issues in how to deliver the ReST course (see Östergren, 2015 [Östergren n.k.a. author CN]).

We made some further refinements in the fourth round (see Vincenti and Zetterberg, 2017). We introduced a “basic ReST exercise” that we used as the only formal homework assignment every week of the course in order to promote overlearning of the most central procedures (for a “basic CMT exercise”, we used a mindfulness of breath exercise adapted from MBSR). The sensory- modality-specific homework exercises were converted to informal exercises that retained their connection to each weeks’ theoretical theme. The first week served as a general introduction to mindfulness. The exercises mainly targeted interoceptive experience and were meant to promote basic familiarity with physiological aspects of stress and relaxation. The second week introduced ideas about effortlessness and direct engagement with experience. The exercises mainly involved exploration of tactile sensations and were meant to illustrate how patience balanced with curiosity can enhance connection with present experience. The third and fourth weeks delved into how thoughts and feelings can contribute to experience without necessarily representing truths or constraints. The exercises mainly involved exploration of auditive and visual aspects of experience and were meant to promote reflexive awareness. The fifth week dealt with the present moment. The exercises involved all senses and allowed greater independence in the practice through extended silences and unguided exploration. (In CMT, a matching progression of exercises and theoretical themes centered on bodily sensations, thoughts, and emotions).

While centering on each week’s theme, the theoretical discussions in ReST also entailed content about management of limited attention resources, including ways to minimize resource expenditure, how to recognize signs of depletion, and the conditions and practices that facilitate restoration. The course contents omitted any reference to training and instead emphasized the ongoing pursuit of a balanced state of mind and activity pattern. The homework instructions also omitted any reference to training the mind and instead emphasized the benefits of regularly stopping usual activities and of connecting with sensory aspects of experience in one’s life. This contrasted with CMT, where we also discussed the management of attention and related mindfulness skills but with a training-based rationale for the management strategies and exercises.

### Previous Findings From Tests of ReST Mechanisms and Acceptability

As mentioned above, the ReST courses were given in four data collection rounds across which the exercises and other aspects of the protocol were successively developed and refined. In each round, university students with stress or concentration problems were randomly assigned to ReST or to a parallel course of CMT. CMT used exercises and an exercise rationale that was adapted from MBSR, and emphasized focused-attention practices. Across all rounds, the ReST classes were held in a university botanical garden, most often a tropical greenhouse, while the CMT classes were held in classroom settings. In two earlier articles, different segments of data from the four rounds have been used to address two sets of concerns that motivated the development of ReST.

#### Does ReST Practice Better Promote Restoration and Improve Restoration Skills?

Studying those participants who went through the entire course, Lymeus et al. (2018) reported results of attention performance tests obtained before and after ReST and CMT practice on weeks 1, 3, and 5 of the courses. Because of methodological improvements in Round 4, data from rounds 1–3 were combined as Study 1 and from Round 4 as Study 2. In Study 1, the ReST and CMT participants completed one test before and after the 90-min classes: the Letter-Digit Substitution Test (LDST; van der Elst et al., 2006) which is sensitive to variations in selective and executive aspects of attention (see e.g., Mirsky et al., 1991). In Study 2, they completed two tests before and after 20-min meditation sessions at the beginning of the classes. In addition to the LDST, they completed a Trail-Making Test (TMT; Reitan, 1958; also see Tombaugh, 2004) where part A involves simple visual search and part B adds a set-switching task. The difference between part A and B scores isolates the performance decrement incurred with the set-switching task (i.e., the switching cost) and so is specifically sensitive to variation in executive attention. The ReST participants meditated with the basic ReST exercise and CMT participants meditated with a conventional analog: a mindfulness of breath meditation adapted from MBSR.

The results showed that across the course weeks, ReST practice was consistently attended by improvement (i.e., restoration) with regard to LDST performance and increasingly attended by improvement with regard to switching cost. Meanwhile, CMT practice was increasingly taxing with regard to LDST performance and did not substantively affect the switching cost. In the last course week, the performance change from before to after meditation differed significantly between ReST and CMT participants in both studies, and in Study 2 for both measures.

Lymeus et al. (2018) also analyzed change across the five course weeks only in the tests obtained before the classes and practice sessions, with a view to generalized improvements in test performance. In Study 1 and with a stronger measurement design in Study 2, ReST and CMT participants showed clear improvements in LDST and TMT part B performance from week 1 to week 3 whereafter performance leveled off. The cumulative benefits of regular and enhanced restoration in effortless exercises were thus similar to the benefits of effortful attention network training. In sum, Lymeus et al. (2018) demonstrated that ReST incurs less effort than CMT and provides restorative short-term benefits as well as generalized attentional improvements.

#### Is ReST More Acceptable as an Introduction to Mindfulness Training for Beginners With Stress and Concentration Problems?

In a second article, Lymeus et al. (2019) analyzed compliance records from the four rounds of ReST and CMT. They confirmed that larger numbers of randomly assigned ReST participants completed the course: 90% compared to 73% in CMT. The course completion rate for CMT was much like that seen in studies of mindfulness-based treatments such as MBSR (Nam and Toneatto, 2016), and the advantage for ReST was small to medium in effect size. Lymeus et al. (2019) also saw that those who completed ReST and CMT largely adhered to the recommended homework practice, completing around seven homework exercises per week on average. However, the ReST participants maintained a steady practice habit over the 5 weeks while the more decimated sample of CMT participants, who were potentially more motivated or capable, completed gradually fewer homework exercises. Again, the advantage for ReST was small to medium in effect size.

In two serial mediation models, Lymeus et al. (2019) were able to account for substantial proportions of the differences in drop-out and homework completion, respectively, through expected relationships between ratings of state mindfulness (curiosity and decentering) and perceived restorativeness (fascination and being away) obtained in connection with the ReST and CMT classes. In the first model, greater perceived restorative quality in the garden setting supported state mindfulness during the meditation, which in turn explained much of the difference in the course completion rate. In the second model, increase across the course weeks in the degree of state mindfulness achieved in the meditation also increased perceived restorative quality, which in turn explained much of the difference in maintenance of the homework completion rate. These mediation tests affirmed the feasibility of the theoretical model behind ReST and illustrated how ReST training could work to reinforce the restorative quality perceived in the meditation setting.

### The Present Study

The previous studies found that ReST successfully promotes restoration and trains restoration skills, and that it is a more acceptable introduction to mindfulness training than CMT for the given study population. However, it remains to be seen whether the less effortful ReST approach can match the more demanding CMT with regard to its effectiveness in conferring long-term improvements. We consider effects in the theoretically and practically relevant domains of dispositional mindfulness, cognitive functioning, and chronic stress. We test for effects across the four data collection rounds, checking and controlling for effects of Round, but also break out data from Round 4 for separate analyses involving contrasts between the most refined version of the ReST course, CMT, and a passive control condition.

Our hypotheses and analyses are grouped under three aims: (1) to establish the average effects of ReST compared with initial values and compared with a passive control condition, (2) to compare the average effects of ReST against those of CMT, and (3) to compare the likelihood of experiencing reliable, individual-level improvement or deterioration with ReST, CMT, and the passive control condition. In addressing aims (2) and (3), we thus use different approaches to determine whether ReST has any meaningful disadvantage compared with CMT with regard to the measured outcomes.

#### Aim 1: Establishing Average Effects

To establish whether ReST and CMT have positive average effects, we study the change scores representing the difference from before to after the course. We first test whether the course participants’ psychological functioning improved on average over the time spent in mindfulness training:

Hypothesis 1: Average improvements compared to values before the course will be evident after the course for both ReST and CMT. We test this hypothesis with separate analyses for dispositional mindfulness (H1a), cognitive functioning (H1b), and chronic stress (H1c).

However, further comparing the outcomes against change seen without any mindfulness training will strengthen confidence that the outcomes are not simply due to uncontrolled factors associated with the passage of time. It will also allow the calculation of between-subjects effect sizes. In the last data collection round, we therefore included a separately recruited, passive control group against which we compare the outcomes of the fully developed ReST course as well as the CMT course:

Hypothesis 2: Average improvements among ReST and CMT participants will be greater than any changes in the passive control group. We test this hypothesis with separate analyses for dispositional mindfulness (H2a), cognitive functioning (H2b), and chronic stress (H2c). Only Round 4 data are included in these analyses.

#### Aim 2: Comparing the Average Effects of ReST Against CMT

We formulated our central research question in terms of the similarity of the outcomes of ReST and CMT. In determining whether the outcomes of ReST are sufficiently similar to those of CMT, we cannot rely on conventional statistical significance tests that address differences rather than similarity. However, we do not have a sufficiently large sample for formal non-inferiority analyses (see Schumi and Wittes, 2011). Instead, we consider that any disadvantages in the outcomes of ReST should be practically negligible and outweighed by the known advantages of ReST over CMT.

To determine a criterion for the acceptable degree of disadvantage for ReST, we draw on the previous finding that compliance was higher with ReST than with CMT with small to medium-sized effects (Lymeus et al., 2019). We further consider the minimal important difference (Revicki et al., 2008). In the absence of previous knowledge about what degree of benefit might be expected with our specific CMT course and in the absence of external criteria for meaningful improvement, we rely on distribution-based approaches. Revicki et al. (2008) indicate that some studies have set a criterion as low as 0.25 standard deviations (SD) of difference, where a difference of 0.2 is conventionally interpreted as a small effect.

Building on the above, we reason that if the average improvement should be smaller with ReST than with CMT, but no more so than with a small effect size, then ReST retains some advantage over CMT by allowing more people to complete the training and access those benefits. Furthermore, the disadvantage will likely be practically negligible for the participants. Note, however, that we do not disavow the possibility that ReST will demonstrate superiority over CMT with regard to the outcomes of interest. Using the change scores, we test this hypothesis:

Hypothesis 3: Any difference in the degree of improvement with ReST and CMT will be no more than small ($\eta_{p}^{2}$ < 0.01) to the disadvantage of ReST. We test this hypothesis with separate analyses for dispositional mindfulness (H3a), cognitive functioning (H3b), and chronic stress (H3c).

#### Aim 3: Comparing the Likelihood of Improvement and Deterioration With ReST Against CMT and Passive Control

To scrutinize the practical utility of ReST, we consider it meaningful to test for any meaningful difference in the likelihood of improvement as well as deterioration compared with CMT and with no intervention. We determine change on the individual level with the RCI (Jacobson and Truax, 1991). The RCI indicates whether a particular participant’s post-intervention score is reliably different from the pre-intervention score, beyond any variability that could be due to measurement error. It thus allows classification of participants as reliably improved, possibly unchanged, or reliably deteriorated. To compare the utility of ReST and CMT, we consider a compound score based on all three outcome measures (i.e., dispositional mindfulness, cognitive functioning, and chronic stress). Reliable deterioration on any of the three outcomes qualifies a participant for classification as deteriorated. Among those who do not deteriorate on any of the outcomes, those who reliably improve on at least one outcome qualify as improved. As in the previous contrasts between ReST and CMT, we consider that any disadvantage for ReST in the likelihood of improvement or deterioration has to be no more than small to be practically negligible and preserve some of the known advantage of ReST over CMT in terms of acceptability. However, we do not disavow the possibility that ReST will demonstrate superiority over CMT. Using the RCI, we test two hypotheses:

Hypothesis 4: Any difference in the likelihood of reliable change with ReST and CMT will be no more than small (**φ** < 0.1) to the disadvantage of ReST. We test this hypothesis with separate analyses for improvement (H4a) and deterioration (H4b).

Hypothesis 5: The likelihood of reliable change with a course in ReST or CMT will be more advantageous than in the passive control group. Specifically, reliable improvement will be more likely than in the passive control group (H5a) and reliable deterioration will be no more likely than in the passive control group (H5b). Only Round 4 data are included in these analyses.

## Materials and Methods

### Design

We ran parallel ReST and CMT courses in four data collection rounds across which ReST was successively developed and refined while CMT was only adjusted to maintain a close match with regard to course and exercise formats. Participants were randomly assigned to ReST or CMT in each round; however, they could not be randomized across the rounds. The overall study design therefore has two between-subjects factors: Course type (ReST, CMT) and Round (1–4). Our aims involved contrasts between the course types across the rounds while checking and controlling for any effects of Round. In Round 4, we allowed for separate analyses by recruiting larger numbers of course participants and additionally including a passive control condition with separately recruited (and so not randomly assigned) participants from the general student population. Although we must assume that these control participants differed in unmeasured ways from the course participants, the control group allowed us to address validity threats from repeated measurements and shared contextual factors (e.g., seasonality, history). In the separate analyses of Round 4 data, we thus have three levels of the between-subjects factor Course type (ReST, CMT, passive control).

The participants provided data on the measures in focus here before and directly after the courses. The study also involved other design features not in focus here (Lymeus et al., 2018, 2019), including a 6-month follow-up with the same measures analyzed here (see Lymeus, 2019).

The ReST classes were held in settings within a botanical garden, as congruent with the ReST practice approach. The CMT classes were held in sparsely furnished indoor settings as is usual for that practice approach. The low level of external stimulation in the indoor settings is presumably congruent with the focused-attention meditation approach targeting internal experiences. Because the design conflates practice and setting, it does not allow conclusions regarding the relative contributions to the outcomes of the practice approach and the setting. Nor does it allow conclusions regarding how either practice approach would have worked in any other type of setting. However, the design does allow practically relevant conclusions regarding the benefits achieved with each of the two bona-fide approaches to mindfulness training, as they would normally be given in a congruent environment.

### Participants and Procedures

#### Recruitment

In each data collection round, we posted flyers in several areas of our university campus, asking for volunteers for a study about mindfulness training. We particularly stated that we sought students with self-perceived stress or concentration problems but no other major health issues and with little or no meditation experience. Volunteers were called to a screening interview that included the MINI International Neuropsychiatric Interview (Lecrubier et al., 1997). Criteria for exclusion were based on Dobkin et al. (2012): We excluded those who indicated a history of neuropsychiatric disorder, psychoses, hypomanic or manic episodes or recurring depression, moderate to severe post-traumatic stress symptoms, serious self-harm, or suicide attempts; and those with any current moderate to severe psychiatric disorders, suicidal ideation, or ongoing psychological or psychiatric treatment.

For the passive control condition in data collection round 4, we approached students in the campus environment asking for volunteers for a study. To be eligible, they had to certify that they had no major health issues and little or no meditation experience.

#### Assignment

Within each data collection round, eligible mindfulness training volunteers who provided informed consent to participate were stratified by gender and randomly assigned to either ReST or CMT. Altogether, 159 participants were assigned. Of them, 152 provided usable pre-course data. Additionally, 29 control group participants who provided informed consent were included in Round 4. Supplementary Figure S1 gives details of participant flow through the recruitment, training, and evaluation phases of the study.

In Round 1, the course participants could be accommodated in one ReST and one CMT course group. In the later rounds, which recruited larger numbers, participants were accommodated in multiple course groups of ≤12 participants. These met on different weekdays. Participants self-selected a course group that fit their schedule and could not switch groups during the course.

Participation in the courses was free of charge. Participants could drop out at any time without facing any further requests or consequences. They were, however, promised three cinema tickets if they completed the course and all measurements in connection with the course. The control group participants were also promised three cinema tickets for completing all measurements.

#### Ethical Review

The research was approved by the regional ethical review board in Uppsala, Sweden (registration number: 2013/033) and adhered to the Declaration of Helsinki.

### ReST and CMT Courses

The courses had one 90-min class each week over five course weeks. Each class included at least two meditation sessions of 15–30 min length, theoretical discussion, and homework consultation. In the homework consultation, instructors gave a rationale and advice for establishing a regular meditation practice. They also gave instructions to practice with one formal and one informal exercise on most of the days leading up to the next class (i.e., in weeks 1–4; no new homework was assigned in the last class). Each formal and informal homework exercise was 15–20 min long. Participants who completed the formal and the informal exercise on four days each course week and additionally participated in all in-class exercises would thus have spent 2 × (17.5 min × 4 times × 4 weeks) + 45 min × 5 classes = 785 min ≈ 13 h in active meditation over the course weeks.

In each data collection round, the ReST and CMT course were matched for structure across the weeks and within the classes, and for the form and amount of assigned homework. An experienced clinical psychologist and mindfulness instructor (FL) led the work and collaborated with different psychologists-in-training (MA, JA, CF, CN, JV, AZ) in providing the courses, participating equally in the ReST and CMT classes. A preliminary script was prepared for each part of the course to ensure quality and consistency (contact the first author to discuss possibilities to use these scripts in replications). An overview of the contents of the ReST and CMT courses in the different rounds of data collection is provided in Supplementary Table S1.

#### Restoration Skills Training

The ReST classes were held in a botanical garden located adjacent to several university campus buildings (Supplementary Figure S2 shows photos of the course settings). Most classes used a tropical greenhouse which in pilot studies had shown high perceived restorative quality compared to different indoor campus settings (i.e., higher ratings of fascination and being away; Kihlberg, 2012; Nordfeldt, 2012). The greenhouse had five rooms with different climate, all richly planted with different types of vegetation. Three rooms had large trees providing overhanging foliage. The greenhouse contained several water bodies, some of which held fish. Tropical frogs also inhabited the greenhouse and contributed to the auditory environment with chirping sounds. Sounds also emanated from the climate control system, and to some extent from the outside. Outdoor areas of the botanical garden and an orangery were also used for some classes. These settings, too, were expected to be high in restorative quality.

In the ReST exercises, the instructors led participants in exploration of experiences emanating from sensory connection with stimuli in the environment, and in embracing all contents in the experiential stream. Some exercises were done sitting and others standing or walking. Many of the exercises transitioned from guided practice to unguided exploration of the environment. The homework assignments did not require participants to meditate in outdoor or natural settings because this could have introduced undue constraints on the practice. Instead, they instructed participants to explore aspects of their setting wherever they were. The homework consultations and theoretical discussions directed participants to pursue greater mindfulness by connecting with sensory aspects of their day-to-day living environments.

#### Conventional Mindfulness Training

The CMT classes were held in sparsely furnished, small classrooms in a campus building adjacent to the botanical garden. The rooms had closed curtains to prevent outside views and no artwork or decorations. The auditory environment was generally quiet, given the late hour of the workday. Building on pilot studies (Kihlberg, 2012; Nordfeldt, 2012), these rooms were expected to be low in both restorative qualities and demands for directed attention and therefore thought to be suitable for focused-attention meditation mainly targeting internal experiences.

For the CMT exercise instructions, we drew on existing instructions for mindfulness of breath-practice, body scan, and other common exercises in MBSR, which we adapted to the briefer format. We selectively included exercises that emphasize focused-attention practice over open-monitoring to preserve the experimental contrast. For most exercises, participants sat in a circle near the center of the room. Some exercises were completed standing or walking around in the same room. The homework assignments instructed participants to find a quiet spot to meditate in stillness with closed eyes and focus on internal experiences. The homework consultations and theoretical discussions directed participants to pursue greater mindfulness by training their capacity for awareness and focus in their day-to-day activities.

### Measures

We measured dispositional mindfulness with a short version of the Five Facet Mindfulness Questionnaire (FFMQ; Baer et al., 2006; Lilja et al., 2011). It has 29 items about how often in the last month a person had experiences of non-judgment, non-reactivity, acting with awareness, observing, and describing. The occurrence of each experience is indicated with a five-point scale (1 = never, 5 = always). After reversal of negatively formulated items, observed internal consistency (Cronbach’s *α*) was = 0.849 before the course and = 0.870 after the course. The score used for analysis was the mean of all item responses, with higher scores indicating higher dispositional mindfulness.

We measured general cognitive functioning with the Cognitive Failures Questionnaire (CFQ; Broadbent et al., 1982). It was presented by Broadbent et al. as an indicator of cognitive vulnerability to stress (see also Kaplan, 1995). It has 25 questions about how often in the last month a person made mistakes in the areas of perception, action, and memory. The occurrence of the mistakes is indicated with a five-point scale (0 = never, 4 = very often). Observed internal consistency was = 0.870 before the course, and = 0.877 after the course. The score used for analysis was the mean of all item responses, with higher scores indicating poorer cognitive functioning.

We measured perceived chronic stress with the Perceived Stress Scale (PSS; Cohen et al., 1983). It measures how often in the last month a person felt unable to handle the demands in their lives. It has 14 questions about experiences of overload, loss of control, and inability to cope. The occurrence of the experiences is indicated with a five-point scale (0 = never, 4 = very often). After reversal of positively formulated items, observed internal consistency was = 0.832 before the course, and = 0.859 after the course. The score used for analysis was the mean of all item responses, with higher scores indicating higher perceived stress.

### Statistical Analyses

The data were analyzed with SPSS 26. We analyzed the data per-protocol, including only completers who provided data both before and after the course, as well as with an intention-to-treat approach. Per-protocol analyses allow straightforward interpretations because they reveal the observed effects of the courses for those who completed them. However, they do not preserve the initial randomization and can bias comparisons when missingness is unequal in the groups (as with the larger drop-out from CMT). As is common in clinical research, we therefore repeated all the hypothesis tests with an intention-to-treat approach that included all course participants who provided data before the course by replacing missing data for those who failed to provide data after the course. Such analyses can preserve the initial randomization but introduce untestable assumptions (Gupta, 2011; White et al., 2011) and can sometimes underestimate differences between intervention groups which is particularly problematic when hypotheses concern similarity (Snapinn, 2000; Schumi and Wittes, 2011). With these complicating factors in mind, we considered that the per-protocol and the intention-to-treat analyses must both yield support for conclusions regarding any advantage for ReST over CMT (see e.g., Snapinn, 2000; Gupta, 2011; Schumi and Wittes, 2011), whereas an indication of a disadvantage for ReST compared to CMT would be accepted even when the analyses yielded inconsistent results.

#### Dealing With Missing Data

The intention-to-treat analyses required us to replace the missing outcome data from the drop-outs. Using the variables obtained before the course as predictors, Little’s MCAR test showed, as anticipated, that the missing outcome data were not missing completely at random [χ^2^(4) = 9776.25, *p* < 0.001]. Multiple imputation was deemed inappropriate and we proceeded with expectation-maximization imputation, aware that the imputed data could be biased to an unknown degree if missing outcome data were related to poorer outcomes. For control group participants in Round 4, however, we had no interest in making any untestable assumptions about their development (cf. White et al., 2011). Rather, we considered the somewhat decimated sample of control participants who provided complete data as most useful for our contrasts, and so excluded those who failed to provide data after the course.

#### Initial Levels and Balance

Before testing our hypotheses, we checked the initial levels and balance between ReST and CMT participants in total and in each data collection round, and between ReST, CMT, and control participants in Round 4. For these analyses, we used a series of univariate ANOVA with Course type as the between-subjects factor.

#### Addressing Aim 1: Establishing Average Effects

To determine the average effects of ReST and CMT, we used a series of ANCOVA that included the initial value obtained before the course for the respective measures as a covariate, and the change score as the dependent variable (as per Vickers and Altman, 2001).

To test whether average improvements compared to values before the course were evident after the course (H1), the analyses included participants from all data collection rounds and used two between-subjects factors: Course type (ReST, CMT) and Round (1–4). We considered a reliable effect to be present when the 95% confidence interval (CI) for the average change scores did not overlap zero.

To test whether average improvements were greater than any changes in the passive control group (H2), we analyzed data from Round 4 with one between-subjects factor: Course type (ReST, CMT, passive control). Here, we based the statistical inference on the 95% CI for pairwise contrasts between the groups. In order to obtain effect size estimates for the contrasts, we also used independent samples *t*-tests on the change scores, from which we calculated *r*.

#### Addressing Aim 2: Comparing the Average Outcomes of ReST Against CMT

To test whether any difference in the degree of improvement with ReST and CMT was no more than small to the disadvantage of ReST (H3), we scrutinized the test statistics from the ANCOVA described above. We checked the effect size estimates for the Course type factor for any effect that exceeded the conventional criterion for a small effect ($\eta_{p}^{2}$ ≥ 0.01).

#### Addressing Aim 3: Comparing the Likelihood of Improvement and Deterioration With ReST Against CMT and Passive Control (H4, H5)

To test whether any difference in the likelihood of reliable change with ReST and CMT was no more than small to the disadvantage of ReST (H4), we used Chi-square analyses on the RCI classifications and the conventional criterion for a small effect (*φ* ≥ 0.1). To obtain RCI classifications, we calculated the RCI for each of the measures as per Jacobson and Truax (1991; also see Wise, 2004). These were based on the test-retest reliability that we observed in the passive control group in Round 4 (for FFMQ *r* = 0.711, CFQ *r* = 0.842, PSS *r* = 0.707), and the observed standard deviations of the course participants’ initial scores on the respective measurement scales (see Table 1). This rendered the RCI for 95% confidence in change as follows: FFMQ = 0.65, CFQ = 0.54, PSS = 0.72. For each measure, participants with change scores larger than the RCI were classified as reliably improved or, if the change was for the worse, reliably deteriorated. Participants with change scores smaller than the RCI were classified as unchanged. To obtain an overall index, we classified each participant as either improved on at least one of the measures, unchanged on all measures, or deteriorated on at least one of the measures. In the few cases where a participant had improved on some measure(s) but also deteriorated on at least one, we classified them as deteriorated. Where the Chi-square analyses indicated an effect that exceeded the criterion (*φ* ≥ 0.1), we proceeded with *post hoc* tests to identify the source of the difference. The *post hoc* tests contrasted improved vs. unchanged and deteriorated participants, and deteriorated vs. unchanged and improved participants.

**TABLE 1:** Descriptive and test statistics for measures obtained before the course, for restoration skills training (ReST) and conventional mindfulness training (CMT) participants in the four data collection rounds (R1–R4) and in total across the rounds.

	Descriptives		Between-subjects contrast			
	*M*	*SD*	*F*	*df*	*p*	$\eta_{p}^{2}$
**FFMQ**						
*Round 1*						
ReST	3.15	0.52	1.34	1,17	0.262	0.073
CMT	2.88	0.46
*Round 2*						
ReST	3.06	0.36	4.56	1,29	0.041	0.136
CMT	2.74	0.46
*Round 3*						
ReST	2.91	0.31	3.06	1,45	0.087	0.064
CMT	3.10	0.43
*Round 4*						
ReST	3.11	0.42	0.02	1,53	0.886	0.000
CMT	3.13	0.45
*Total*						
ReST	3.04	0.40	0.18	1,15	0.668	0.001
CMT	3.01	0.47
**CFQ**						
*Round 1*						
ReST	1.64	0.36	0.10	1,17	0.754	0.006
CMT	1.72	0.58
*Round 2*						
ReST	1.92	0.52	0.85	1,29	0.365	0.028
CMT	1.77	0.39
*Round 3*						
ReST	1.81	0.55	0.07	1,45	0.794	0.002
CMT	1.85	0.57
*Round 4*						
ReST	1.78	0.50	1.15	1,53	0.288	0.021
CMT	1.93	0.52
*Total*						
ReST	1.80	0.50	0.27	1,15	0.607	0.002
CMT	1.85	0.52
**PSS**						
*Round 1*						
ReST	1.94	0.52	0.10	1,17	0.761	0.006
CMT	1.87	0.40
*Round 2*						
ReST	1.99	0.61	0.30	1,29	0.590	0.010
CMT	2.10	0.49
*ROUND 3*						
ReST	2.06	0.45	1.65	1,45	0.206	0.035
CMT	1.88	0.48
*Round 4*						
ReST	1.92	0.51	0.05	1,53	0.816	0.001
CMT	1.89	0.46
*Total*						
ReST	1.98	0.51	0.46	1,15	0.500	0.003
CMT	1.93	0.47

*The measurement scale for FFMQ is 1–5, with higher scores indicating higher dispositional mindfulness, and for CFQ and PSS it is 0–4 with higher scores indicating more problems. Univariate ANOVA conducted separately for each Round and for the total sample with Course type (ReST, CMT) as a between-subjects factor.*

To test whether reliable improvement was more likely than in the passive control group (H5a) and reliable deterioration was no more likely than in the passive control group (5b), we subjected RCI classifications from Round 4 to Chi-square analysis which included ReST, CMT, and passive control participants. With regard to improvement, we looked for statistically significant differences to proceed with planned *post hoc* tests. With regard to deterioration, we proceeded with *post hoc* tests even for simple numerical differences to address any possibility of a disadvantage for ReST or CMT. The *post hoc* tests were done as described above but separately for ReST vs. control, CMT vs. control, and ReST vs. CMT.

## Results

### Initial Levels and Balance

Table 1 shows descriptive statistics for the initial 152 course participants (ReST *n* = 75, CMT *n* = 77) who comprise the intention-to-treat sample. The average ratings with FFMQ, CFQ, and PSS were near the center of the respective scales; respondents tended to answer “sometimes” with regard to how often in the last month they had different mindfulness experiences, experienced cognitive failures, and felt unable to handle the demands in their lives. For the total sample, the ratings were highly similar for ReST and CMT participants. However, the FFMQ ratings showed unexpected variation between the randomly assigned ReST and CMT participants in Rounds 2 and 3. In Round 4, the passive control group participants provided ratings before the course that were fairly similar to those of ReST and CMT participants: FFMQ *M* = 3.23 (SD = 0.40), CFQ *M* = 1.59 (0.42), PSS *M* = 1.81 (SD = 0.52).

### Aim 1: Establishing Average Effects

#### Hypothesis 1

Of the 113 participants who completed the courses, 105 (93%; ReST *n* = 56, CMT *n* = 49) also completed the assessments after the course and could be included in the completer sample in analyses for Hypothesis 1. Table 2 shows estimated marginal means (adjusted for Round and pre-test score) and CIs for the change seen with the courses. Our main concern is for the CIs for the total of ReST and CMT participants, which we hypothesized would not overlap zero. Data for each data collection round are also provided for transparency; note, however, that the individual rounds are not powered to detect effects in the small range.

**TABLE 2:** Estimated marginal means (M) and confidence intervals (CI) for the change scores representing the difference from before to after the restoration skills training (ReST) and conventional mindfulness training (CMT) courses in ratings with the Five Facet Mindfulness Questionnaire (FFMQ), Cognitive Failures Scale (CFQ), and Perceived Stress Scale (PSS).

	Completer sample			Intention-to-treat sample		
	*M*	95% CI		*M*	95% CI	
**FFMQ**						
*Round 1*						
ReST	0.18	–0.08	0.44	0.18	–0.02	0.38
CMT	0.53	0.23	0.82	0.43	0.24	0.62
*Round 2*						
ReST	0.36	0.12	0.60	0.33	0.18	0.48
CMT	0.26	0.05	0.46	0.25	0.09	0.41
*Round 3*						
ReST	0.31	0.13	0.49	0.29	0.17	0.42
CMT	0.27	0.07	0.47	0.27	0.15	0.39
*Round 4*						
ReST	0.32	0.16	0.47	0.30	0.19	0.42
CMT	0.35	0.17	0.52	0.32	0.21	0.43
*Total*						
ReST	0.29	0.19	0.40	0.28	0.20	0.35
CMT	0.35	0.24	0.46	0.32	0.24	0.39
**CFQ**						
*Round 1*						
ReST	0.04	–0.24	0.32	0.00	–0.22	0.22
CMT	–0.52	–0.84	–0.19	–0.41	–0.62	–0.20
*Round 2*						
ReST	–0.42	–0.68	–0.15	–0.35	–0.52	–0.19
CMT	–0.12	–0.34	0.11	–0.15	–0.32	0.02
*Round 3*						
ReST	–0.24	–0.43	–0.05	–0.27	–0.40	–0.13
CMT	–0.26	–0.48	–0.04	–0.27	–0.41	–0.14
*Round 4*						
ReST	–0.34	–0.51	–0.17	–0.33	–0.46	–0.20
CMT	–0.27	–0.46	–0.08	–0.28	–0.41	–0.16
*Total*						
ReST	–0.24	–0.35	–0.12	–0.24	–0.32	–0.15
CMT	–0.29	–0.41	–0.17	–0.28	–0.36	–0.20
**PSS**						
*Round 1*						
ReST	0.21	–0.09	0.50	0.17	–0.07	0.40
CMT	–0.17	–0.51	0.17	–0.18	–0.40	0.05
*Round 2*						
ReST	–0.28	–0.56	0.01	–0.24	–0.42	–0.07
CMT	0.02	–0.21	0.25	0.00	–0.18	0.18
*Round 3*						
ReST	–0.12	–0.32	0.09	–0.13	–0.28	0.01
CMT	–0.17	–0.40	0.07	–0.18	–0.32	–0.04
*Round 4*						
Rest	–0.41	–0.59	–0.23	–0.37	–0.50	–0.23
CMT	–0.44	–0.65	–0.24	–0.35	–0.48	–0.22
*Total*						
ReST	–0.15	–0.27	–0.03	–0.14	–0.23	–0.06
CMT	–0.19	–0.32	–0.06	–0.18	–0.26	–0.09

*The marginal means were estimated in ANCOVA that included Course type (ReST, CMT) and Round (1–4) as between-subjects factors and the respective pretest scores as a covariate.*

Average improvements in FFMQ ratings (i.e., positive values) and in CFQ and PSS ratings (i.e., negative values) compared to values before the course were evident for both ReST and CMT participants, for both the completer and ITT samples, affirming our hypotheses regarding effects on dispositional mindfulness (H1a), cognitive functioning (H1b), and chronic stress (H1c).

#### Hypothesis 2

In Round 4, the completer sample comprised 22 ReST participants, 17 CMT participants, and 21 control participants. The ITT sample comprised 27 ReST participants, 28 CMT participants, and the 21 control participants. Table 3 shows estimated marginal means and CIs for the contrasts between the degree of change seen from before to after ReST, CMT, and the control condition in Round 4. Our main concern is for the CIs for the differences in change from before to after the course between ReST and control participants and between CMT and control participants, which we hypothesized would not overlap zero. Both ReST and CMT participants’ average improvements in FFMQ (i.e., positive values) and in CFQ and PSS (i.e., negative values) ratings were greater than the changes in the control group, for both the completer and ITT samples, affirming our hypotheses regarding effects on dispositional mindfulness (H2a), cognitive functioning (H2b), and chronic stress (H2c). ReST and CMT participants did not differ from each other on any of the outcomes.

**TABLE 3:** Pairwise contrasts between restoration skills training (ReST), conventional mindfulness training (CMT), and passive control (PC) condition participants in data collection round 4, of scores representing change from before the course to directly after the course on the Five Facet Mindfulness Questionnaire (FFMQ), Cognitive Failures Questionnaire (CFQ), and Perceived Stress Scale (PSS).

	Completer sample				Intention-to-treat sample			
	*M*_DIFF_	*p*	*95% CI*		*M*_DIFF_	*p*	*95% CI*	
**FFMQ**								
ReST – PC	0.26	0.010	0.07	0.45	0.25	0.003	0.09	0.41
CMT – PC	0.28	0.009	0.07	0.48	0.26	0.002	0.10	0.42
ReST – CMT	–0.02	0.849	–0.22	0.18	–0.02	0.825	–0.13	0.17
**CFQ**								
ReST – PC	–0.32	0.008	–0.56	–0.09	–0.30	0.003	–0.49	–0.10
CMT – PC	–0.25	0.046	–0.50	–0.01	–0.25	0.013	–0.45	–0.05
ReST – CMT	–0.07	0.569	–0.31	0.17	–0.05	0.621	–0.23	0.14
**PSS**								
ReST – PC	–0.30	0.030	–0.57	–0.03	–0.26	0.029	–0.49	–0.03
CMT – PC	–0.32	0.028	–0.61	–0.04	–0.24	0.042	–0.46	–0.01
ReST – CMT	0.02	0.874	–0.26	0.31	–0.02	0.848	–0.23	0.19

*The marginal means were estimated in ANCOVA that included Course type (ReST, CMT, PC) as a between-subjects factor and the respective pretest scores as a covariate.*

Table 4 shows *t*-test results and effect size estimates for the contrasts between ReST and CMT, respectively, and passive controls. All effects (*r*) were medium to large (range 0.30–0.58).

**TABLE 4:** Independent samples *t*-test results and effect size (*r*) estimates for contrasts between restoration skills training (ReST) and conventional mindfulness training (CMT) against passive control (PC) condition participants in data collection round 4, of scores representing change from before the course to directly after the course on the Five Facet Mindfulness Questionnaire (FFMQ), Cognitive Failures Questionnaire (CFQ), and Perceived Stress Scale (PSS).

	Completer sample				Intention-to-treat sample			
	*t*	*df*	*p*	*r*	*t*	*df*	*p*	*r*
**FFMQ**								
ReST – PC	2.89	41	0.006	0.40	2.81	46	0.007	0.38
CMT – PC	3.36	36	0.002	0.48	3.91	47	<0.001	0.49
**CFQ**								
ReST – PC	3.19	41	0.003	0.44	3.01	46	0.004	0.40
CMT – PC	3.45	36	0.001	0.49	4.92	47	<0.001	0.58
**PSS**								
ReST – PC	2.39	41	0.021	0.34	2.14	46	0.038	0.30
CMT – PC	2.40	36	0.022	0.37	2.30	47	0.026	0.32

### Aim 2: Comparing the Effects of ReST and CMT

The analyses for Hypothesis 3 build on the same samples and analyses used in testing Hypothesis 1, described above.

#### Hypothesis 3

Table 5 shows additional test statistics from the ANCOVA performed in Step 1, which included Course type, Round, and pretest score as predictors of the degree of improvement from before to after the 5-week courses. Having a poorer pretest score on the given measure (lower for FFMQ, higher for CFQ and PSS) was strongly related to improvement ($\eta_{p}^{2}$ range: 0.266–0.387, *p*’s < 0.001). For FFMQ, the average improvement was large (completer $\eta_{p}^{2}$ = 0.405, ITT $\eta_{p}^{2}$ = 0.463; *p*’s < 0.001) and it was not significantly different across the Rounds. For CFQ, the average improvement was medium to large (completer $\eta_{p}^{2}$ = 0.139, ITT $\eta_{p}^{2}$ = 0.224; *p*’s ≤ 0.001). However, CFQ improvement varied across rounds, as reflected in the significant Course × Round interaction term. As seen in Table 2, the ReST participants failed to show reliable improvement in Round 1 while CMT participants failed to show improvement in Round 2. For PSS, the average improvement was medium to large (completer $\eta_{p}^{2}$ = 0.190, ITT $\eta_{p}^{2}$ = 0.253; *p*’s < 0.001), but for ReST and CMT participants alike, it was only reliable across both the completer and ITT samples in Round 4.

**TABLE 5:** ANCOVA test statistics for explanation of change from before to after the two mindfulness training courses in ratings with the Five Facet Mindfulness Questionnaire (FFMQ), Cognitive Failures Scale (CFQ), and Perceived Stress Scale (PSS) by Course type (restoration skills training vs. conventional mindfulness training), data collection round of the study (Round 1–4), and the respective pretest scores, for the intention-to-treat sample and completer sample.

	Completer sample			Intention-to-treat sample		
	*F*	*p*	$\eta_{p}^{2}$	*F*	*p*	$\eta_{p}^{2}$
**FFMQ**						
Corrected Model (df = 8)	6.82	<0.001	0.363	13.01	<0.001	0.421
Intercept (df = 1)	65.35	<0.001	0.405	123.15	<0.001	0.463
Pretest (df = 1)	47.47	<0.001	0.331	90.39	<0.001	0.387
Course type (df = 1)	0.54	0.464	0.006	0.62	0.432	0.004
Round (df = 3)	0.54	0.945	0.004	0.08	0.972	0.002
Course type × Round (df = 3)	0.54	0.315	0.036	1.24	0.296	0.025
**CFQ**						
Corrected Model (df = 8)	6.40	<0.001	0.348	12.93	<0.001	0.420
Intercept (df = 1)	15.56	<0.001	0.139	41.35	<0.001	0.224
Pretest (df = 1)	34.84	<0.001	0.266	85.39	<0.001	0.374
Course type (df = 1)	0.38	0.537	0.004	0.49	0.485	0.003
Round (df = 3)	0.14	0.935	0.004	0.47	0.701	0.010
Course type × Round (df = 3)	3.29	0.024	0.093	3.49	0.018	0.068
**PSS**						
Corrected Model (df = 8)	6.91	<0.001	0.365	11.92	<0.001	0.400
Intercept (df = 1)	22.53	<0.001	0.190	48.53	<0.001	0.253
Pretest (df = 1)	34.79	<0.001	0.266	73.76	<0.001	0.340
Course type (df = 1)	0.20	0.654	0.002	0.27	0.604	0.002
Round (df = 3)	5.22	0.002	0.140	6.33	<0.001	0.117
Course type × Round (df = 3)	1.83	0.146	0.054	2.75	0.045	0.055

*For Course, CMT = 0 and ReST = 1. Round = 1–4. Completer N = 105 (error term degrees of freedom = 96), intention-to-treat N = 152 (error term degrees of freedom = 143).*

However, our main concern is for the effect size estimates for the Course type factor, which we hypothesized would be no more than small to the disadvantage of ReST compared with CMT. The effect sizes for the effect of Course type on FFMQ, CFQ, and PSS were all $\eta_{p}^{2}$ < 0.01 (n.s.), for both the completer and ITT samples. The degree of improvement was thus similar with ReST and CMT, affirming our hypotheses with regard to dispositional mindfulness (H3a), cognitive functioning (H3b), and chronic stress (H3c).

### Aim 3: Comparing the Likelihood of Improvement and Deterioration With ReST Versus CMT and Passive Control

The analyses for Hypotheses 4 and 5 build on the same samples used in testing Hypothesis 1.

#### Hypothesis 4

Table 6 shows frequencies and Chi-square test statistics for the contrasts between ReST and CMT participants in the likelihood of reliable change with the courses. Of the 105 participants who completed the ReST and CMT courses, forty (38%) improved reliably. Of the 152 participants who were included in the ITT sample, 52 (34%) improved reliably. However, reliable deterioration was seen in seven participants (7% of the completer sample). The effect sizes for the contrasts between the ReST and CMT distributions in change classifications were *φ* < 0.1 for both the completer and ITT samples, and thus did not give reason to proceed with *post hoc* tests. The likelihood of reliable change was thus similar with ReST and CMT, affirming H4a (regarding improvement) and H4b (regarding deterioration).

**TABLE 6:** Classification plots and Chi-square test statistics for the number of restoration skills training (ReST) and conventional mindfulness training (CMT) participants who improved, remained unchanged, and deteriorated from before to directly after the course, based on reliable change index scores.

	Completer sample		Intention-to-treat sample	
	ReST	CMT	ReST	CMT
Improved	19 (21.3)	21 (18.7)	23 (25.7)	29 (26.3)
Unchanged	33 (30.9)	25 (27.1)	48 (45.9)	45 (47.1)
Deteriorated	4 (3.7)	3 (3.3)	4 (3.5)	3 (3.5)
	χ^2^(2) = 0.88, *p* = 0.673*, *φ* = 0.092		χ^2^(2) = 0.91, *p* = 0.686*, *φ* = 0.077	

Expected frequencies are given in parentheses. *Denotes a p-value derived from Fisher’s Exact Test due to small expected values in some cells.

#### Hypothesis 5

Table 7 shows observed frequencies and Chi-square test statistics for the contrasts between ReST, CMT, and passive control participants in Round 4 in the likelihood of reliable change with the courses. Of the 39 participants who completed the ReST and CMT courses in Round 4, 18 (46%) were reliably improved compared to two (10%) of the 21 passive control participants. Of the 55 participants who were included in the Round 4 ITT sample, 21 (38%) were reliably improved. However, reliable deterioration was seen in two course participants (5% of the completer sample) and 2 passive control participants (10%). The change distributions differed significantly between the groups for both the completer and ITT samples. The *post hoc* tests performed with the completer sample confirmed that ReST participants were more likely than control participants to improve [χ^2^(1, *N* = 43) = 9.92, Exact *p* = 0.003, *φ* = 0.480] and no more likely than control participants to deteriorate [χ^2^(1, *N* = 43) < 0.01, Exact *p* = 1.00, *φ* = 0.007]. However, CMT participants did not differ significantly from control participants in their likelihood of improvement [χ^2^(1, *N* = 38) = 3.75, Exact *p* = 0.107, *φ* = 0.314]. They were no more likely than control participants to deteriorate [χ^2^(1, *N* = 38) = 1.71, Exact *p* = 0.492, *φ* = 0.212 to the advantage of CMT]. The conclusions were the same in *post hoc* tests with the ITT sample. For ReST, reliable improvement was thus more likely than in the passive control group, affirming H5a, and reliable deterioration was no more likely than in the passive control group, affirming H5b. For CMT, the analyses only support H5b.

**TABLE 7:** Classification plots and Chi-square test statistics for the number of restoration skills training (ReST), conventional mindfulness training (CMT), and passive control (PC) participants who improved, remained unchanged, and deteriorated from before to directly after the course in data collection round 4, based on reliable change index scores.

	Completer sample			Intention-to-treat sample		
	ReST	CMT	PC	ReST	CMT	PC
Improved	12 (7.3)	6 (5.7)	2 (7.0)	12 (8.2)	9 (8.5)	2 (6.4)
Unchanged	8 (13.2)	11 (10.2)	17 (12.6)	13 (17.4)	19 (18.1)	17 (13.5)
Deteriorated	2 (1.5)	0 (1.1)	2 (1.4)	2 (1.4)	0 (1.5)	2 (1.1)
	χ^2^(4) = 11.79, *p* = 0.007*, *φ* = 0.443			χ^2^(4) = 9.30, *p* = 0.026*, *φ* = 0.350		

Expected frequencies are given in parentheses. *Denotes a p-value derived from Fisher’s Exact Test due to small expected values in some cells.

## Discussion

Taking the 5-week ReST course conferred benefits for the average participant, with regard to dispositional mindfulness and cognitive functioning. Regarding chronic stress, the benefits were less consistent in this study, emerging as reliable only with the last iteration of the ReST course. Looking at change on the individual level, over one-third of those who completed ReST enjoyed reliable improvement in their psychological functioning. The likelihood of improvement with ReST was substantially greater than among passive controls, and the likelihood of deterioration was small and no greater than among passive controls. In our conservative comparisons against a CMT course, we could not identify any meaningful disadvantage for ReST.

In Round 4, both ReST and CMT had medium to large average effects compared with the passive controls. Our interventions were thus not less effective than the average for MBSR in controlled studies with non-patient samples (Eberth and Sedlmeier, 2012; Sedlmeier et al., 2018). Our CMT comparison condition thus largely worked as expected, conferring benefits with regard to dispositional mindfulness and cognitive functioning. As with ReST, its effects on chronic stress were inconsistent. In contrast to ReST, however, the likelihood of improvement with CMT was not reliably greater than among passive controls.

We conclude that ReST is efficacious and safe with regard to the measured domains of psychological functioning. Any possible disadvantages of ReST compared with the conventional approach are small enough to be outweighed by the advantages of ReST in terms of its higher acceptability (Lymeus et al., 2019) and short-term restorative effects (Lymeus et al., 2018). Any possible disadvantages are also likely to be practically negligible for the participants. Although we do not measure skill acquisition directly (a complex matter: see e.g., Grossman, 2008; Sauer et al., 2013; Abdoun et al., 2019), the results are coherent with theory and previous findings that suggest that the ReST approach to mindfulness training can teach skills that have broad relevance for adaptation. Importantly, ReST improves psychological functioning without relying on participants’ willingness and ability to practice with effortful focused-attention exercises, as conventional mindfulness courses for beginners typically do.

### Strengths and Limitations

We have compared ReST against a bona-fide, formally comparable alternative treatment. Such comparisons are rare in meditation training studies (Davidson and Kaszniak, 2015). Furthermore, the comparison was done with experimental methods applied in field conditions. This allows practically relevant conclusions regarding the relative merits of ReST and CMT. However, our CMT course was not a strict replication of any established mindfulness course, so we cannot directly generalize to MBSR or any other specific course package. Rather, our CMT was an application of the proposed dominant mechanism behind early improvements in psychological functioning with mindfulness training: attention network training through effortful focused attention practice. Our results have implications for the validity of that proposition.

Our contrasts between ReST, CMT, and the passive control condition in Round 4 bolster confidence that any effects seen with the mindfulness training are not only artifacts of shared external factors in the population or setting. Because the control participants were recruited separately, however, the passive condition is an imperfect counterfactual: the control participants were from the same general population but had not sought mindfulness training so they might have developed differently from the course participants in some respects even if the course participants had not undergone training.

We recruited university students not merely for convenience but because we view them as people engaged in a challenging occupation who might better manage the particular demands of their work if they acquire the skills we taught in the courses. Using otherwise healthy students who experience stress or concentration problems in trying to meet the high cognitive demands in their daily lives, we consider our sample a fair representation of large segments of the modern, urban population. When they volunteered for the study, the participants were unaware that they could be assigned to mindfulness training in a garden setting, so the benefits of ReST are not exclusive to people who are specifically attracted to nature-based interventions. However, because the participants volunteered for a study about mindfulness training, we cannot assume that either ReST or CMT would work well for people who would not spontaneously seek mindfulness training. We also cannot assume that ReST would work similarly for people living and working under different conditions, with other sociodemographic characteristics, or with any specific disorders or disabilities. Furthermore, the study was not conducted as a clinical trial: Replication with full adherence to clinical trial principles could extend the scope of conclusion to a health care context.

We advertised for participants who experienced stress or concentration problems, but we conducted no tests to ascertain the objective presence of such problems before inclusion. We saw that the sample included participants with varying initial values on CFQ and PSS, including those with poorer values; however, the average initial scores were not high in some absolute sense. We also saw that those participants with more initial problems tended to improve more. The inclusion of participants with fewer initial problems would therefore have constrained the ability to detect average improvements; our tests of average effects may therefore be considered conservative.

The drop-out rate from CMT was similar to the average in mindfulness treatment studies, and high enough to pose a problem for the evaluation of its outcomes (Nam and Toneatto, 2016; also see Lymeus et al., 2019), We could not obtain outcome data from the course drop-outs because we had promised them at enrollment that they could drop out without facing any further requests. The imputed data estimates improvements among the drop-outs based on observed improvements among the completers. It is, however, unlikely that those who left the course had improved or would have continued to improve comparably as those who completed it. The ITT analyses could therefore have overestimated the benefits of CMT. We thus consider the ITT analyses as a quite stringent test of how ReST compares with CMT.

We rely on retrospective, self-reported outcome assessments. The FFMQ, CFQ, and PSS are all established and valid measures of foundational aspects of real-life functioning (Cohen, 1988; Sauer et al., 2013; Carrigan and Barkus, 2016). However, each of these measures has been debated (see e.g., Grossman, 2008; Nielsen et al., 2016; Könen and Karbach, 2018), and so have retrospective self-report assessments more generally (e.g., Shiffman et al., 2008). For cognitive functioning, the outcomes we report here are consistent with the trajectory of development in repeated performance test data reported by Lymeus et al. (2018). Inclusion of more direct approaches to measuring mindfulness and stress could possibly have achieved greater precision and confidence in the achieved functional improvements. It could also have been relevant to assess potential mediators of outcomes such as psychological flexibility (Kashdan and Rottenberg, 2010) and self-compassion (Neff and Dahm, 2015).

### Implications for Practice and Research

Otherwise healthy individuals with stress or concentration problems can enjoy improvements in dispositional mindfulness and cognitive functioning through ReST. With ReST, the average participant can expect no more risk and no less benefit in any of the measured domains than with a more demanding conventional approach to mindfulness training. ReST is therefore a viable alternative to CMT for people who struggle under heavy demands in their daily lives. It remains to be determined how long and to what degree these benefits are sustained after completion of the course: 6-month follow-up data will be published in a separate article (also see Lymeus, 2019).

Given that ReST works through low-effort practices supported by restorative nature experience, the findings of benefits that are similar to those achieved with CMT cast further doubt on the assumption that attention network training is a key mechanism behind the improvements in psychological functioning seen in early stages of mindfulness training (cf. Lutz et al., 2008; Tang et al., 2015). Instead, much of the early improvement with conventional approaches to mindfulness training might occur during a short initial honeymoon phase when the meditation experience may be dominated by restorative processes (Lymeus et al., 2018, 2019 and Lymeus, 2019 discuss the involved processes at length). When beginners learn to self-regulate the meditative state, practice may become more dominated by attention network training processes, and therefore more effortful. It would be at this stage that compliance problems could ensue for participants who have insufficient access to the needed cognitive resources.

The spread of experiences of distraction and stress in modern society is feasibly connected to increasing intensity and constancy of demands on our limited cognitive resources (Kaplan, 1995; Kaplan and Berman, 2010). Many of those who seek mindfulness training may pursue respite from demands and renewal of a sense of presence lost in efforts to maintain focus and composure while completing the many tasks associated with modern work and living. These individuals may not be well served by focused-attention practice in the early stages of mindfulness training, which might instead exacerbate fatigue and cause frustration (Lymeus et al., 2018; Baer et al., 2019). In the monastic settings where mindfulness training was first developed and formalized, people may have entered into and engaged with the training under quite different premises, more supportive of effortful pursuits.

Environmental approaches to the management of adaptive resources have the advantage over individual, skill-based approaches that they mitigate fatigue and reinstate lost capacities without imposing further demands. This study is part of a larger effort to study the users, and the potential uses and benefits of a historic botanical garden that spatially and organizationally is part of the Uppsala University. Studies in other university campus settings have indicated that access to natural features and settings can help mitigate the stress of student life and potentially boost learning outcomes (Felsten, 2009; Hipp et al., 2015; van den Bogerd et al., 2018). On the other hand, skill-based approaches have the advantage that they can train adaptive capabilities that then can be deployed at later times and even in unsupportive contexts. Studies on mindfulness-based interventions for students have, for instance, shown improved ability to handle the stress of taking an exam (e.g., Shapiro et al., 1998; Shearer et al., 2016). ReST joins the advantages of the environmental and skill-based approaches, drawing on restorative processes supported by natural environments while also teaching widely applicable skills.

We cannot disentangle the effects of the ReST practices as such from those of the natural practice setting in the present design. However, we note that ReST appeared to perform relatively poorly in our first data collection round, when the ReST course was most similar to the CMT course. When we progressed with the development of a practice approach more fit to the environment, we saw better outcomes. We propose that adaptations of the conventional approach to mindfulness training are necessary to achieve a working congruence between the practice and a natural meditation setting. To advance understanding of these issues, we have initiated further research to disentangle effects of the practice and the setting (see Shamsaee, 2016 and Bergsten, 2020 for some preliminary findings).

## Data Availability Statement

The datasets generated for this study are available via the Swedish National Data Service (https://doi.org/10.5878/p34t-9j15).

## Ethics Statement

The studies involving human participants were reviewed and approved by Swedish Ethical Review Authority. The patients/participants provided their written informed consent to participate in this study.

## Author Contributions

FL designed the study, led the work to develop and teach the ReST and CMT courses, and led the data collection under the supervision of TH and PL. In different data collection rounds, MA, JA, CF, CN, JV, and AZ contributed to the development of course materials and the collection of data, and performed preliminary analyses on subsets of the data under the supervision of TH and FL. The manuscript was drafted by FL building in part on unpublished Master of Science theses by MA, JA, CF, CN, JV, and AZ. Those MSc theses each covered different subsets of the study and were completed under the supervision of TH and FL. Critical revisions were provided by TH and PL. All authors contributed to the article and approved the submitted version.

## Conflict of Interest

The authors declare that the research was conducted in the absence of any commercial or financial relationships that could be construed as a potential conflict of interest.
