# Supplementary material for: Mindfulness-Based Restoration Skills Training (ReST) in a Natural Setting Compared to Conventional Mindfulness Training: Psychological Functioning After a Five-Week Course
Source: Front Psychol. 2020 Aug 12;11:1560. doi: 10.3389/fpsyg.2020.01560 (PMC7438830; doi:10.3389/fpsyg.2020.01560)
Supplement: Supplementary file 1 [file Data_Sheet_1.pdf]

Supplement to Lymeus et al. Mindfulness-based restoration skills training (ReST) in a natural setting compared to conventional mindfulness training: Psychological functioning after a five-week course

Table S1. Overview of the restoration skills training (ReST) and conventional mindfulness training (CMT) courses as they were given in the four rounds of the study.

		Restoration skills training (ReST)			Conventional mindfulness training (CMT)		
	Week	Theme	In-class exercises	Homework	Theme*	In-class exercises	Homework
Round 1	1	Introduction<br>Setting: Orangery	1. Raisin exercise<br>2. Sitting meditation<br>3. Body scan	Formal: Inward and outward attention<br>Informal: Mindful washing	Introduction	1. Raisin exercise<br>2. Sitting meditation<br>3. Body scan	Formal: Inward and outward attention<br>Informal: Mindful washing
	2	Attention<br>Setting: Greenhouse	1. Walking meditation<br>2. Mindfulness of sound<br>3. Body scan	Formal: Internal and external attention<br>Informal: Mindfulness in routine activity	Attention	1. Counting breaths<br>2. Mindfulness of sound<br>3. Body scan	Formal: Internal and external attention<br>Informal: Mindfulness in routine activity
	3	Acceptance<br>Setting: Greenhouse	1. Mindful movement<br>2. Loving kindness	Formal: Internal and external attention<br>Informal: Mindfulness in routine activity	Acceptance	1. Mindful movement<br>2. Loving kindness	Formal: Internal and external attention<br>Informal: Mindfulness in routine activity
	4	The thinking brain<br>Setting: Coniferous grove	1. Loving kindness<br>2. Thoughts as clouds<br>3. Mindful movement	Formal: Body scan<br>Informal: Mindfulness wherever you are	The thinking brain	1. Loving kindness<br>2. Thoughts as clouds<br>3. Mindful movement	Formal: Body scan<br>Informal: Mindfulness wherever you are
	5	Mindfulness of feelings<br>Setting: Kitchen garden, greenhouse	1. Walking meditation<br>2. Mindfulness of feelings<br>3. Mindful movement		Mindfulness of feelings	1. Mindful movement<br>2. Mindfulness of feelings<br>3. Loving kindness	
Round 2	1	Introduction / The body<br>Setting: Greenhouse	1. Raisin exercise and sitting meditation<br>2. Mindfulness of the body, sitting and walking	Formal: Body scan<br>Informal: Mindful walking	Introduction / The body	1. Raisin exercise and sitting meditation<br>2. Body scan	Formal: Body scan<br>Informal: Mindful washing
	2	Curiosity / Touch<br>Setting: Greenhouse	1. Guided tactile meditation<br>2. Guided anchoring and independent tactile exploration	Formal: Tactile meditation<br>Informal: Mindful touching	Attention / The breath	1. Mountain meditation<br>2. Mindfulness of breath	Formal: Body scan<br>Informal: Mindfulness in routine activity
	3	Acceptance / Hearing<br>Setting: Greenhouse	1. Guided sound meditation<br>2. Guided anchoring and independent sensory exploration	Formal: Sound meditation<br>Informal: Mindful hearing	Acceptance / Compassion	1. Mindful movement<br>2. Loving kindness	Formal: One thing at a time<br>Informal: Mindfulness in coming and leaving
	4	The thinking brain / Seeing<br>Setting: Greenhouse	1. Guided visual meditation<br>2. Guided anchoring and independent sensory exploration	Formal: Visual meditation<br>Informal: Mindful seeing	The thinking brain / Thoughts	1. Walking meditation<br>2. Thoughts as clouds	Formal: Passing thoughts<br>Informal: Mindfulness in unpleasant situations

	5	The moment / All experience together<br>Setting: Greenhouse	1. Guided meditation on smell and taste<br>2. Guided anchoring and independent sensory exploration		The moment / Mindfulness of feelings	1. Mindfulness of thoughts and feelings<br>2. Mindfulness of the moment	
	1	Introduction / The body<br>Setting: Greenhouse	1. Raisin exercise and sitting meditation<br>2. Guided body scan and independent walking meditation	Formal: Body scan<br>Informal: Mindful walking	Introduction / The body	1. Raisin exercise and sitting meditation<br>2. Body scan	Body scan
	2	Curiosity / Touch<br>Setting: Greenhouse	1. Guided tactile exploration<br>2. Guided anchoring and independent tactile exploration	Formal: Tactile exploration<br>Informal: Mindfulness of touch	Attention / The breath	1. Mountain meditation<br>2. Mindfulness of breath	Formal: Mindfulness of breath<br>Informal: Mindfulness in routine activity
Round 3	3	Thinking and judging / Hearing<br>Setting: Greenhouse	1. Guided sound meditation<br>2. Guided anchoring and independent auditive exploration	Formal: Auditive exploration<br>Informal: Mindfulness of sound	Acceptance / Feelings	1. Self-compassion<br>2. Walking meditation	Formal: One thing at a time<br>Informal: Mindfulness in unpleasant situations
	4	Thinking and judging / Seeing<br>Setting: Greenhouse	1. Guided visual meditation<br>2. Guided anchoring and independent visual exploration	Formal: Visual meditation<br>Informal: Mindfulness of sight	Detachment / Thoughts	1. Thoughts as clouds<br>2. Mindfulness of thoughts and feelings	Formal: Passing thoughts<br>Informal: Mindfulness in transition between activities
	5	The moment / All experience together<br>Setting: Greenhouse	1. Meditation on smell and taste<br>2. Guided anchoring and independent sensory exploration		The moment / All inner experience together	1. This moment<br>2. Sitting meditation with all internal experience	
	1	Introduction / The body<br>Setting: Greenhouse	1. Basic ReST exercise<br>2. Standing and walking meditation	Formal: Basic ReST exercise<br>Informal: Walking meditation	Introduction / The body	1. Basic CMT exercise<br>2. Body scan	Formal: Basic CMT exercise<br>Informal: Mindfulness of the body
	2	Exploring experience / Touch<br>Setting: Greenhouse	1. Guided tactile meditation<br>2. Guided anchoring and independent tactile exploration	Formal: Basic ReST exercise<br>Informal: Mindfulness of touch	Attention and distraction / The breath	1. Mountain meditation<br>2. Mindfulness of breath	Formal: Basic CMT exercise<br>Informal: Mindfulness of breathing
Round 4	3	Embracing experience / Hearing<br>Setting: Greenhouse	1. Basic ReST exercise<br>2. Guided anchoring and independent auditive exploration	Formal: Basic ReST exercise<br>Informal: Mindfulness of sound	Acceptance and decentering, part 1 / Thoughts	1. Basic CMT exercise<br>2. Thoughts as clouds	Formal: Basic CMT exercise<br>Informal: Mindfulness of thinking
	4	Letting go / Vision<br>Setting: Greenhouse	1. Guided visual meditation<br>2. Guided anchoring and independent visual exploration	Formal: Basic ReST exercise<br>Informal: Mindfulness of sight	Acceptance and decentering, part 2 / Feelings	1. Thoughts and feelings<br>2. Walking meditation	Formal: Basic CMT exercise<br>Informal: Stop and accept
	5	The moment / All experience together<br>Setting: Greenhouse	1. Basic ReST exercise<br>2. Guided anchoring and independent sensory exploration		The moment / All inner experience together	1. Basic CMT exercise<br>2. The moment	

Note. The setting for all CMT classes was class rooms in a university campus building
