# Supplementary material for: Mindfulness-Based Restoration Skills Training (ReST) in a Natural Setting Compared to Conventional Mindfulness Training: Psychological Functioning After a Five-Week Course
Source: Front Psychol. 2020 Aug 12;11:1560. doi: 10.3389/fpsyg.2020.01560 (PMC7438830; doi:10.3389/fpsyg.2020.01560)
Supplement: Supplementary file 2 [file Data_Sheet_2.pdf]

Supplement to Lymeus et al. Mindfulness-based restoration skills training (ReST) in a natural setting compared to conventional mindfulness training: Psychological functioning after a five-week course

Table S2. Descriptive statistics for measures obtained after the course, for restoration skills training (ReST) and conventional mindfulness training (CMT) participants in the four data collection rounds (R1 – R4) and in total across the rounds, and for the passive control group that participated in Round 4 only.

			Completer sample			Intention-to-treat sample		
			n	M	SD	n	M	SD
FFMQ	Round 1	ReST	8	3.23	0.36	9	3.26	0.35
		CMT	6	3.51	0.57	10	3.39	0.46
	Round 2	ReST	9	3.38	0.48	16	3.37	0.37
		CMT	13	3.13	0.30	15	3.16	0.29
	Round 3	ReST	17	3.28	0.42	23	3.27	0.36
		CMT	13	3.30	0.38	24	3.33	0.31
	Round 4	ReST	22	3.34	0.43	27	3.37	0.40
		CMT	17	3.40	0.39	28	3.39	0.31
	Course participants Total	ReST	56	3.32	0.42	75	3.33	0.37
		CMT	49	3.31	0.40	77	3.33	0.33
	Round 4 Passive Controls		21	3.18	0.35	21	3.18	0.35
CFQ	Round 1	ReST	8	1.78	0.49	9	1.73	0.47
		CMT	6	1.25	0.43	10	1.36	0.39
	Round 2	ReST	9	1.50	0.43	16	1.52	0.36
		CMT	13	1.65	0.30	15	1.65	0.29
	Round 3	ReST	17	1.56	0.60	23	1.55	0.52
		CMT	13	1.53	0.48	24	1.56	0.40
	Round 4	ReST	22	1.50	0.42	27	1.47	0.39
		CMT	17	1.52	0.54	28	1.59	0.44
	Course participants Total	ReST	56	1.56	0.49	75	1.54	0.44
		CMT	49	1.52	0.46	77	1.57	0.40
	Round 4 Passive Controls		21	1.67	0.43	21	1.67	0.43
PSS	Round 1	ReST	8	1.76	0.52	9	2.11	0.53
		CMT	6	2.21	0.48	10	1.74	0.40
	Round 2	ReST	9	2.07	0.49	16	1.73	0.52
		CMT	13	1.77	0.67	15	2.02	0.48
	Round 3	ReST	17	1.75	0.44	23	1.87	0.42
		CMT	13	1.83	0.48	24	1.74	0.38
	Round 4	ReST	22	1.49	0.46	27	1.57	0.38
		CMT	17	1.56	0.41	28	1.57	0.39
	Course participants Total	ReST	56	1.74	0.51	75	1.76	0.47
		CMT	49	1.77	0.52	77	1.73	0.43
	Round 4 Passive Controls		21	1.77	0.66	21	1.77	0.66

Note. The measurement scale for FFMQ is 1 – 5, with higher scores indicating higher dispositional mindfulness, and for CFQ and PSS it is 0 – 4 with higher scores indicating more problems.
