# Supplementary material for: Mindfulness-Based Restoration Skills Training (ReST) in a Natural Setting Compared to Conventional Mindfulness Training: Psychological Functioning After a Five-Week Course
Source: Front Psychol. 2020 Aug 12;11:1560. doi: 10.3389/fpsyg.2020.01560 (PMC7438830; doi:10.3389/fpsyg.2020.01560)
Supplement: Supplementary file 3 [file Data_Sheet_3.PDF]

Supplement to Lymeus et al. Mindfulness-based restoration skills training (ReST) in a natural setting compared to conventional mindfulness training: Psychological functioning after a five-week course

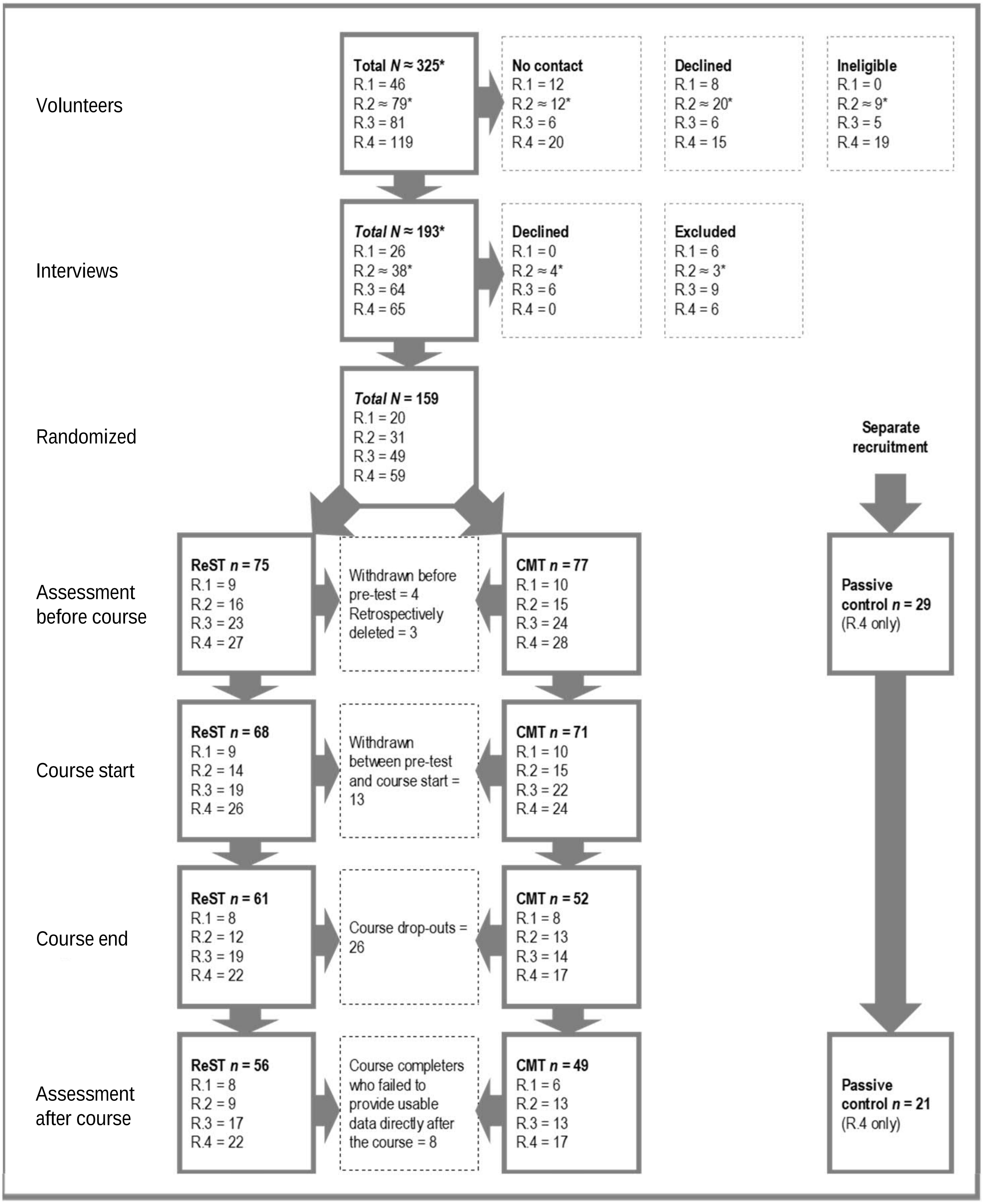

Note. * denotes approximations necessitated by incompleteness of the records from early stages of the recruitment for data collection round 2.

Figure S1. Participant flow through the recruitment, interventions, and evaluation in the four data collection rounds of the study.
