# Supplementary material for: Mindfulness-Based Restoration Skills Training (ReST) in a Natural Setting Compared to Conventional Mindfulness Training: Psychological Functioning After a Five-Week Course
Source: Front Psychol. 2020 Aug 12;11:1560. doi: 10.3389/fpsyg.2020.01560 (PMC7438830; doi:10.3389/fpsyg.2020.01560)
Supplement: Supplementary file 4 [file Data_Sheet_4.PDF]

Supplement to Lymeus et al. Mindfulness-based restoration skills training (ReST) in a natural setting compared to conventional mindfulness training: Psychological functioning after a five-week course

Figure S2a. Study settings

Indoor settings

(examples from one of several similar class-rooms used)

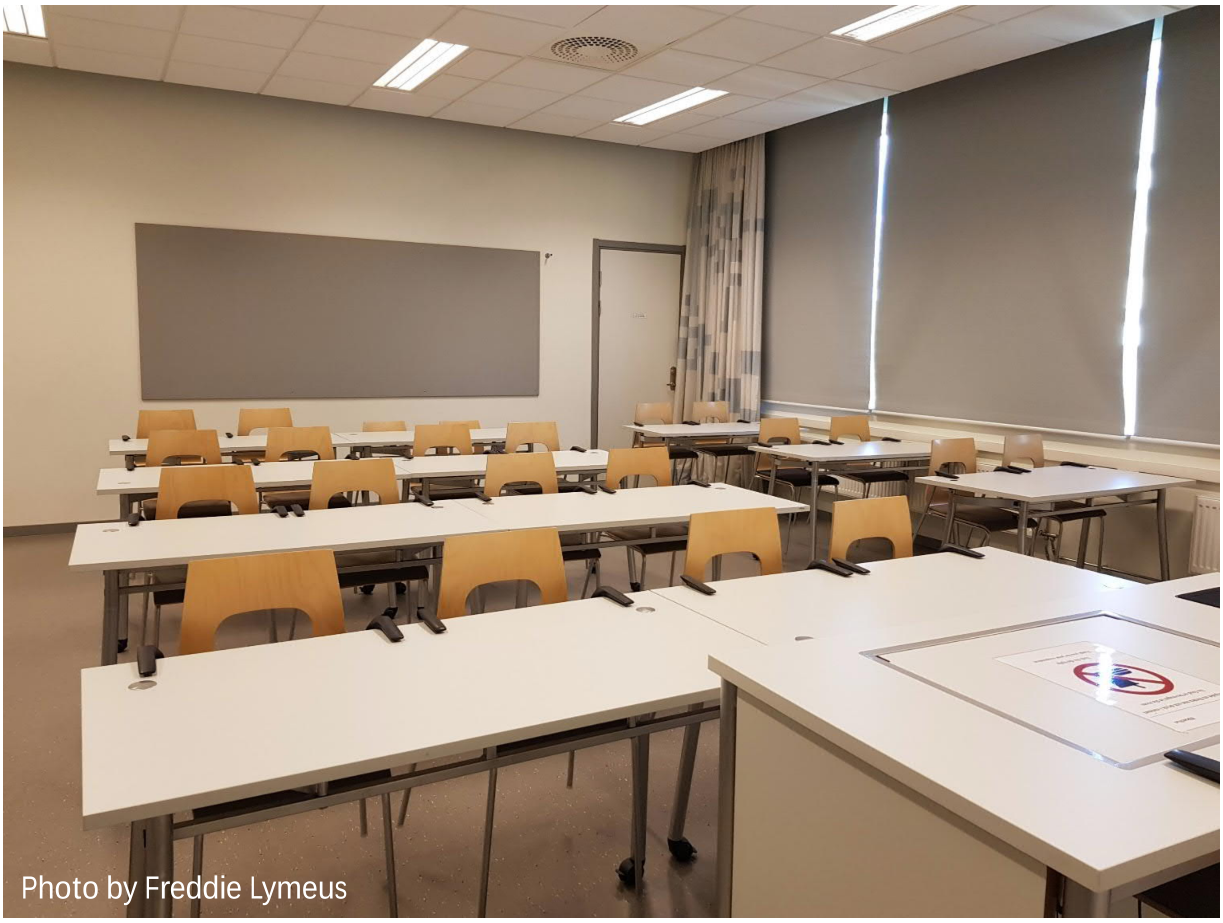

Original class-room arrangement

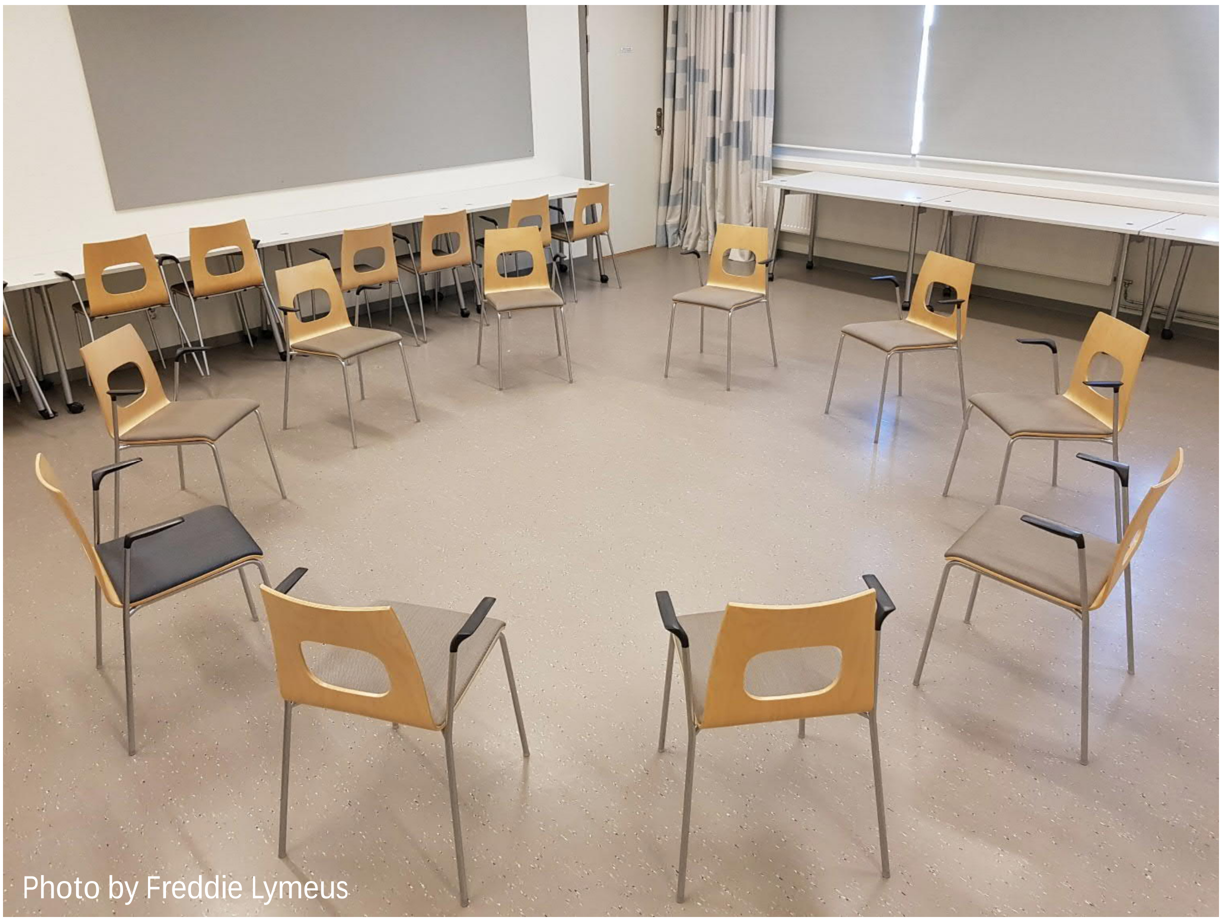

Arrangement for CMT classes

Figure S2b. Study settings

Relationship between campus building used for indoor settings and tropical greenhouse

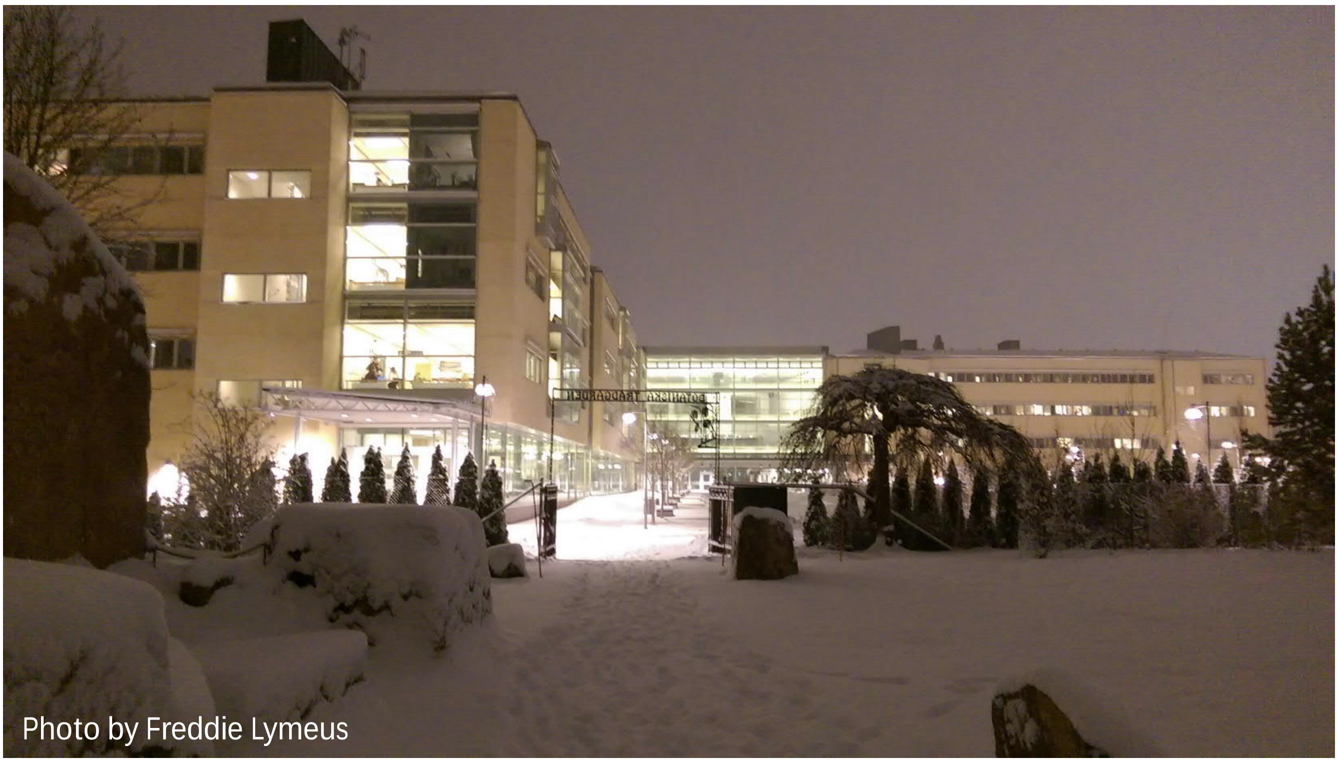

View north-east from the center-point of the connecting path

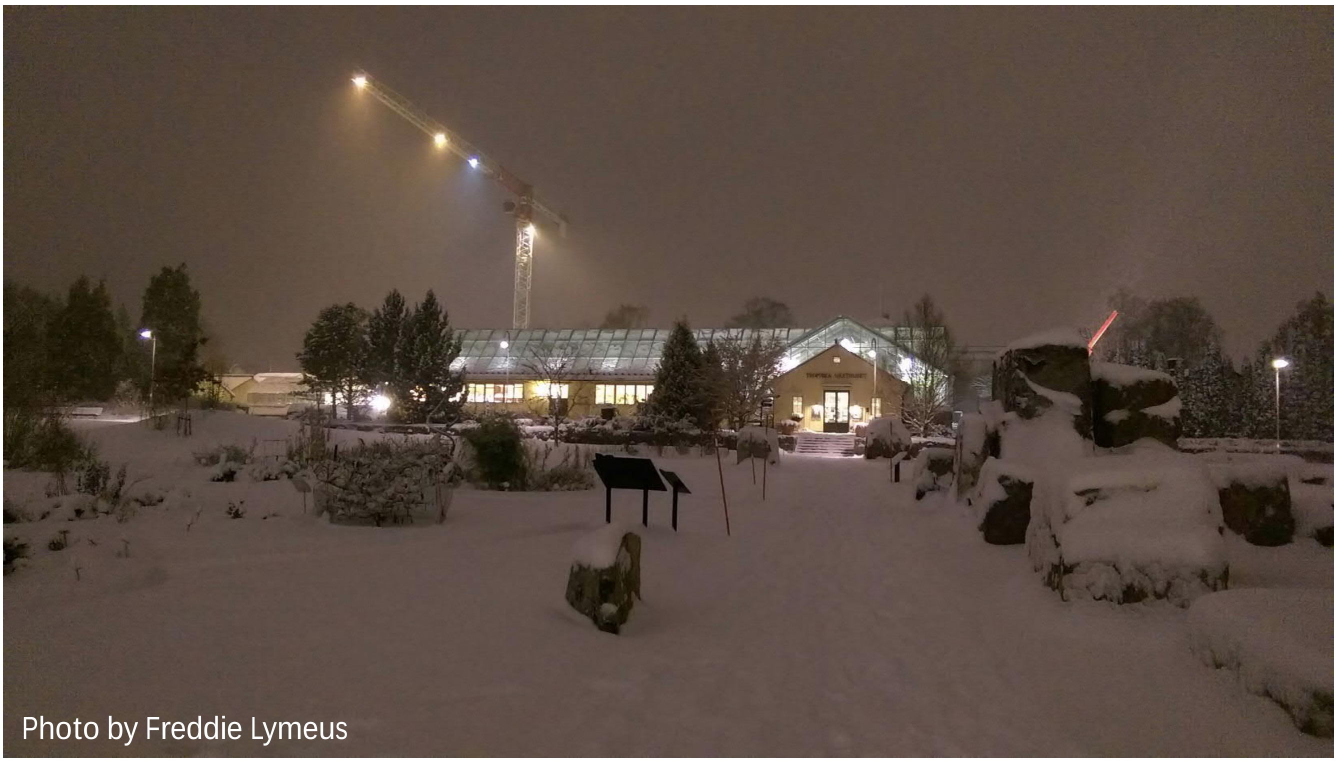

View south-west of the connecting path, taken from garden gates

Supplement to Lymeus et al. Mindfulness-based restoration skills training (ReST) in a natural setting compared to conventional mindfulness training: Psychological functioning after a five-week course

Figure S2c. Study settings

Tropical greenhouse interior scenes
1. "Victoria pond"

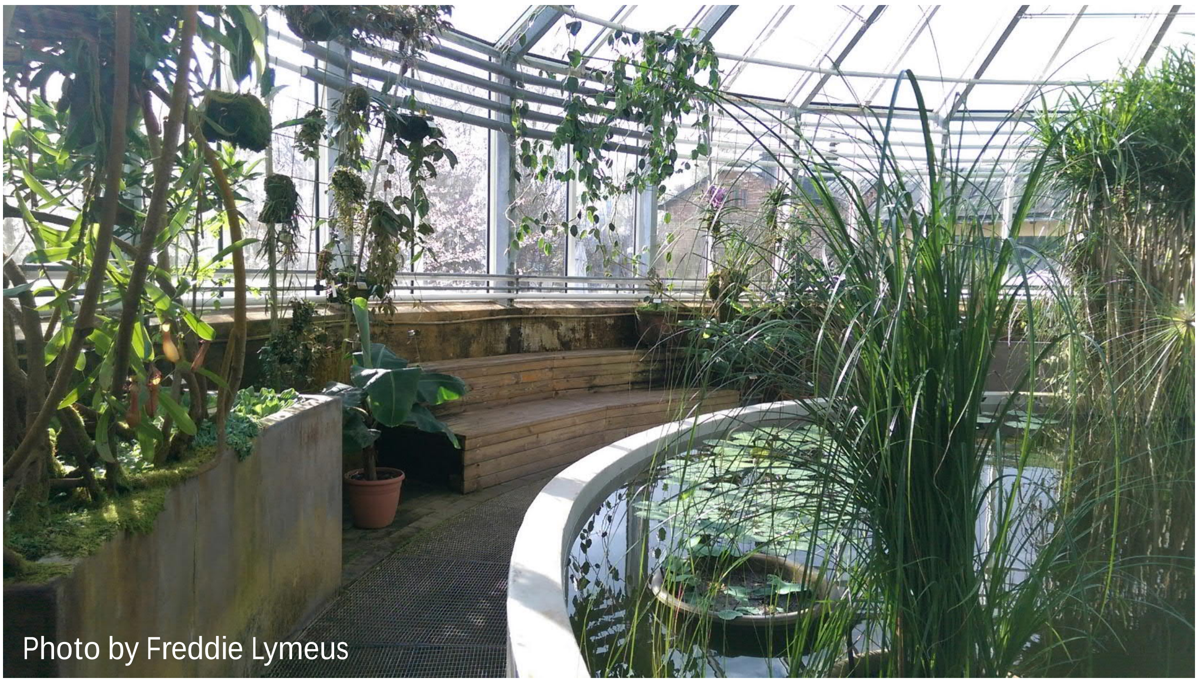

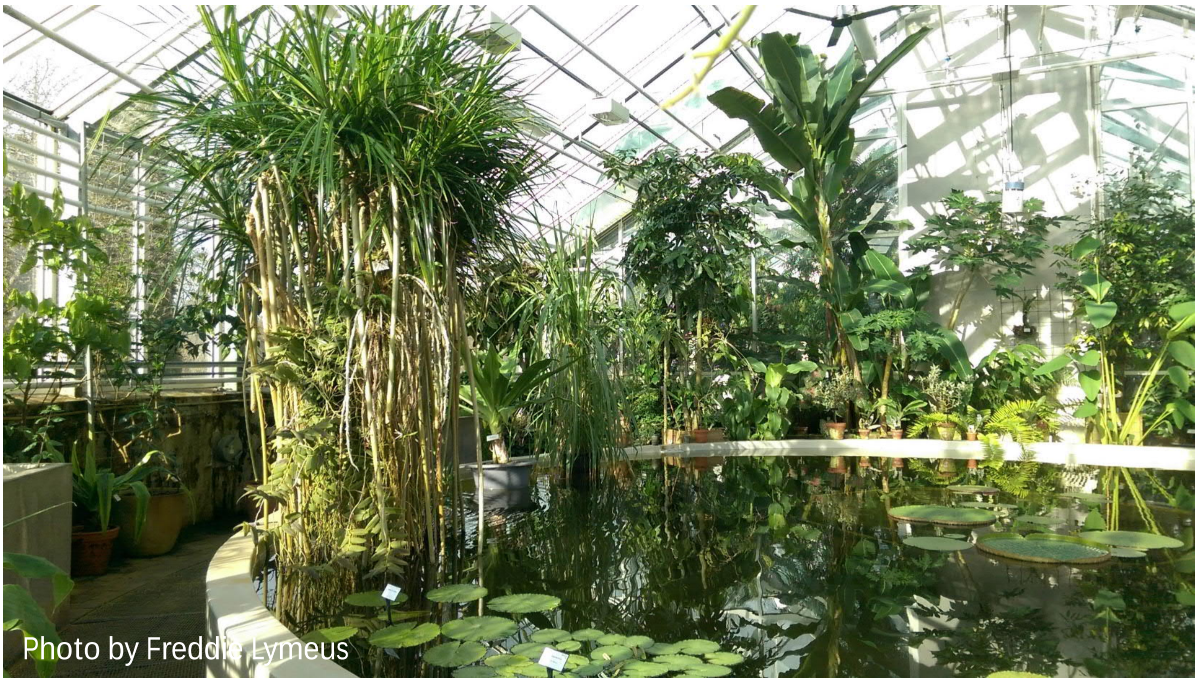

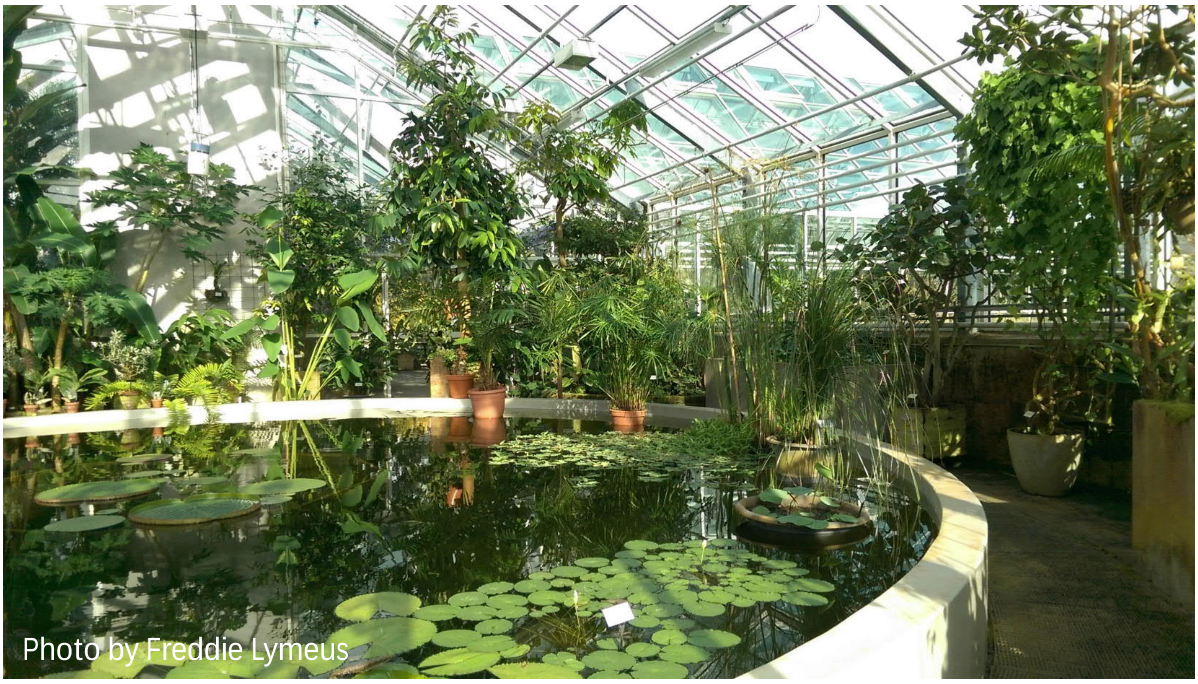

Scenes from the Victoria room, including bench used for some seated ReST exercises

Supplement to Lymeus et al. Mindfulness-based restoration skills training (ReST) in a natural setting compared to conventional mindfulness training: Psychological functioning after a five-week course

Figure S2d. Study settings

Tropical greenhouse interior scenes
2. ”Winter garden”

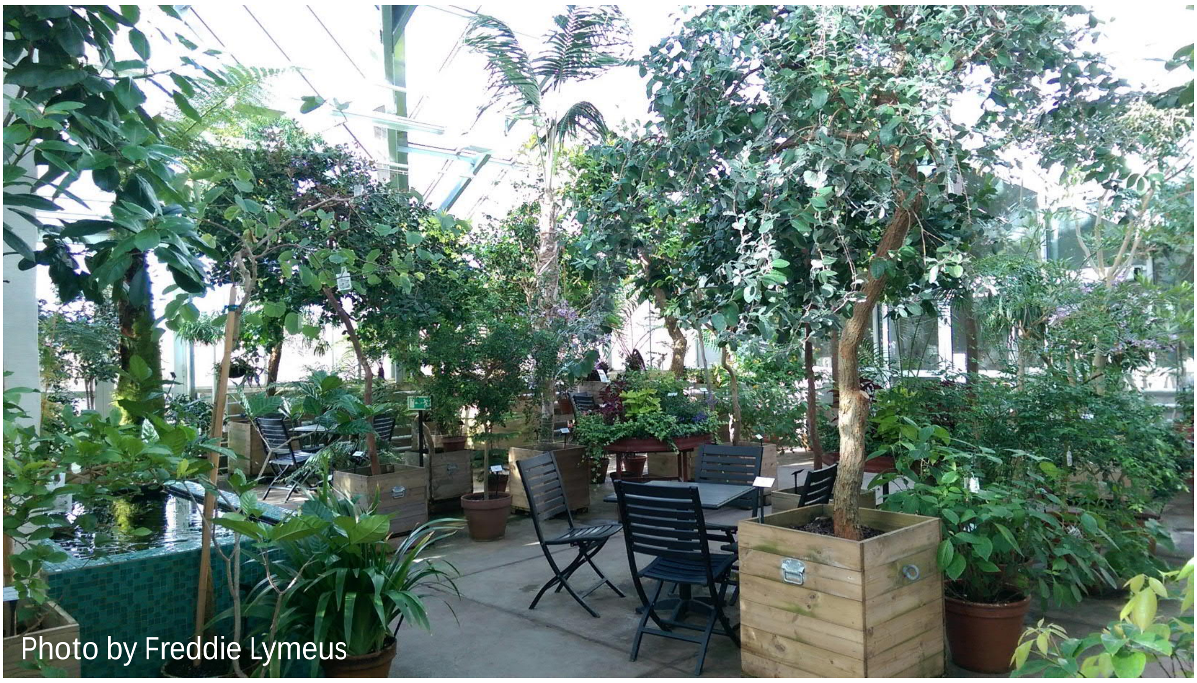

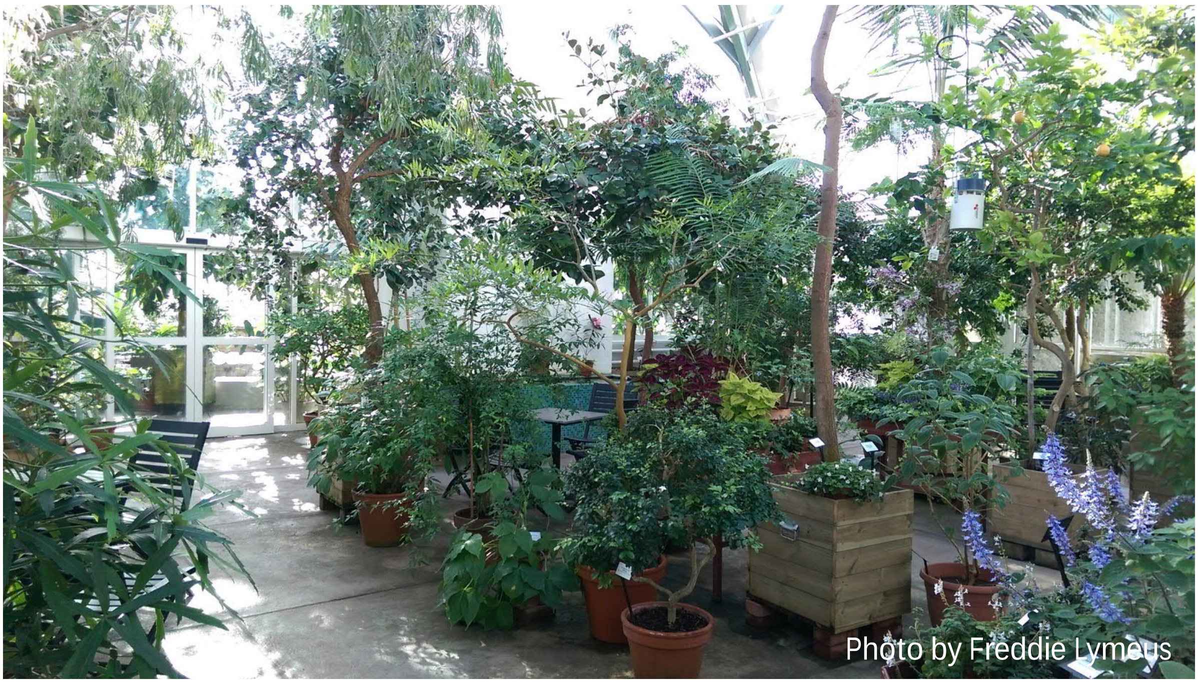

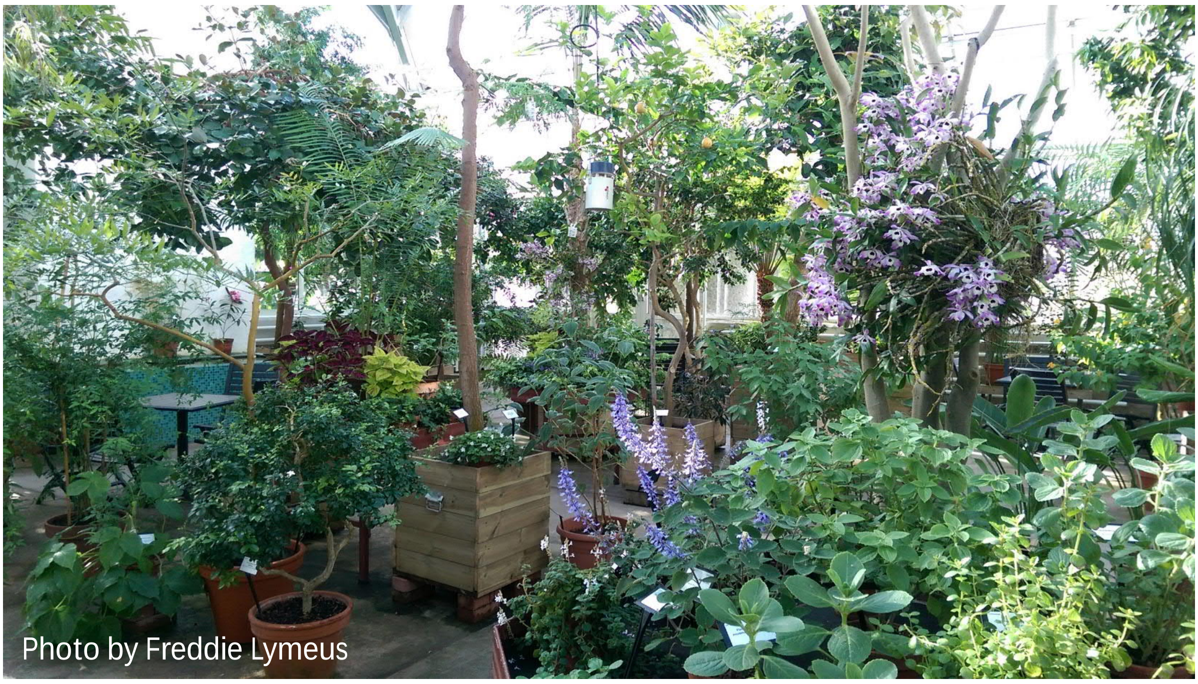

Scenes from the Winter Garden room, including furniture used in some seated ReST exercises

Supplement to Lymeus et al. Mindfulness-based restoration skills training (ReST) in a natural setting compared to conventional mindfulness training: Psychological functioning after a five-week course

Figure S2e. Study settings

Tropical greenhouse interior scenes
3. ”Rain Forest”

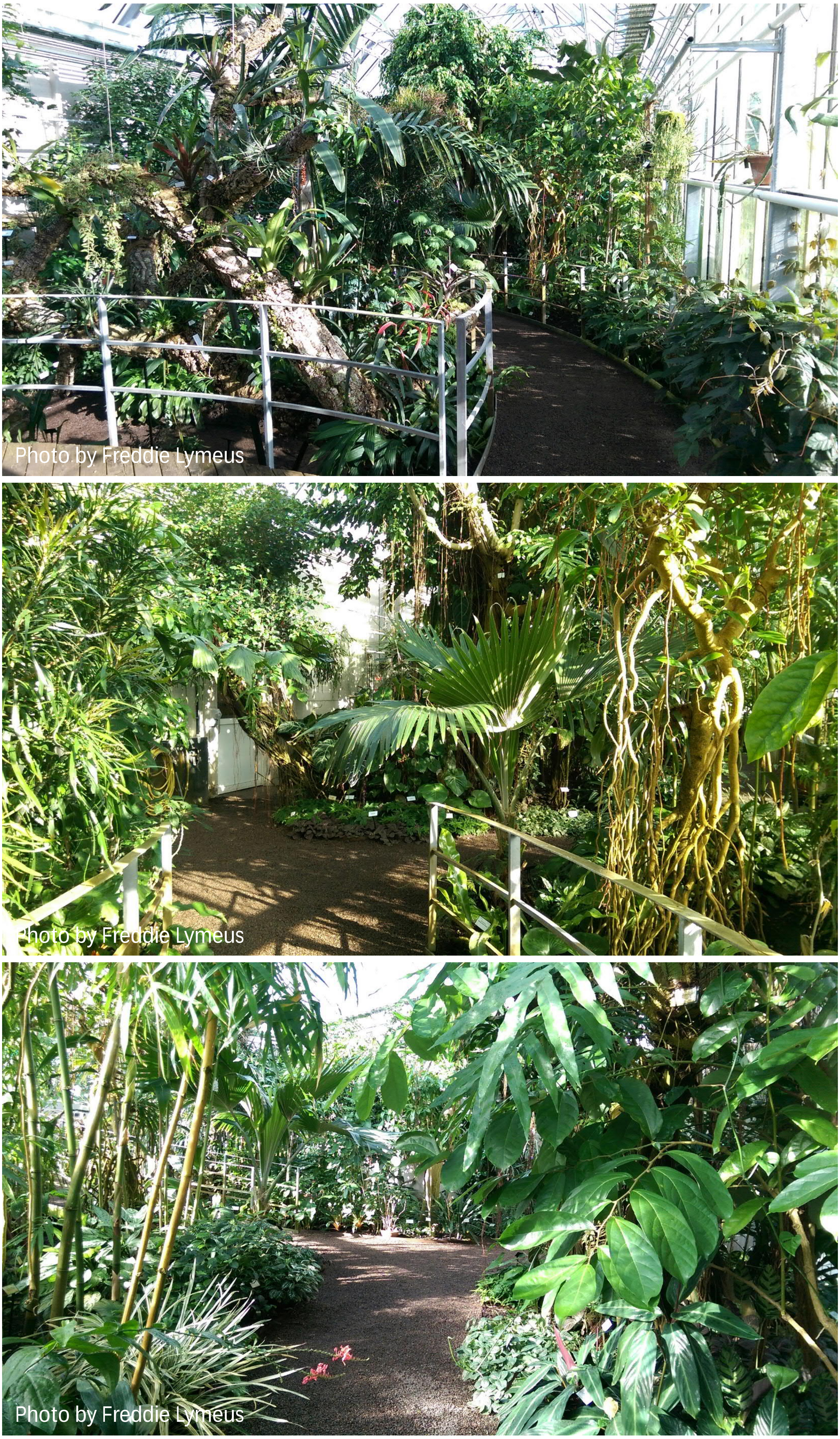

The Rain Forest room, that participants could enter during independent exercises

Supplement to Lymeus et al. Mindfulness-based restoration skills training (ReST) in a natural setting compared to conventional mindfulness training: Psychological functioning after a five-week course

Figure S2f. Study settings

<u>Tropical greenhouse interior scenes</u>
4. Other greenhouse settings

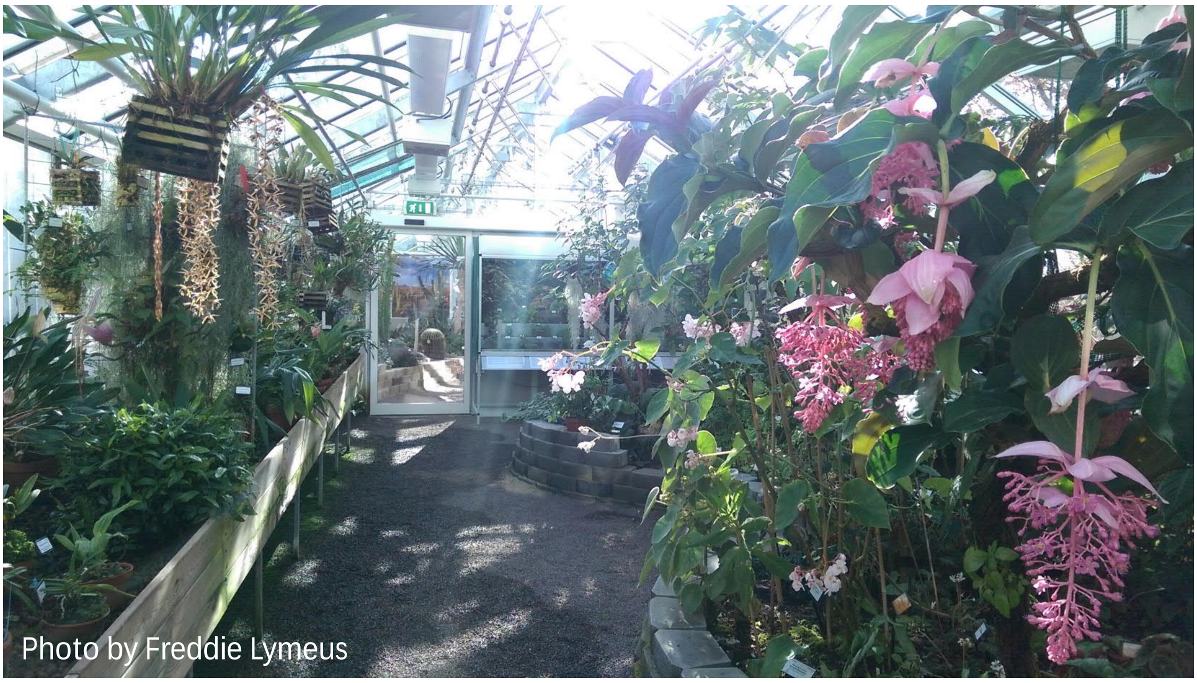

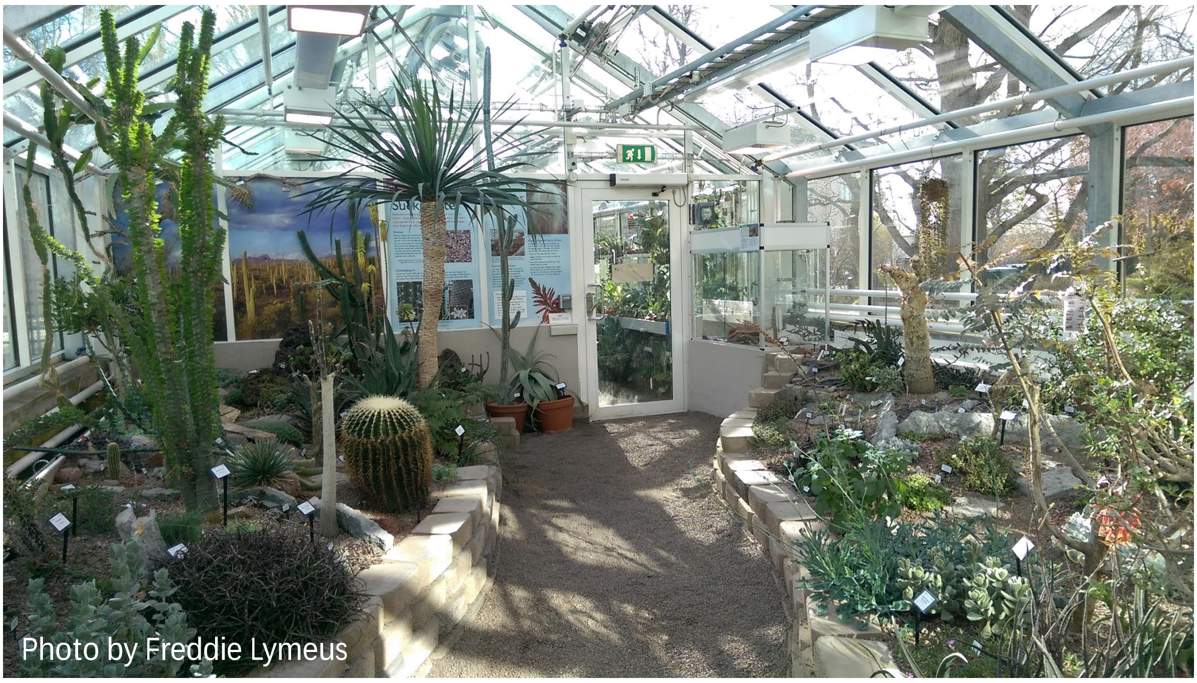

Orchid room and succulent room, that participants
could enter during independent exercises
